# Molecular Genetics Reveal That Silvatic *Rhodnius prolixus* Do Colonise Rural Houses

**DOI:** 10.1371/journal.pntd.0000210

**Published:** 2008-04-02

**Authors:** Sinead Fitzpatrick, Maria Dora Feliciangeli, Maria J. Sanchez-Martin, Fernando A. Monteiro, Michael A. Miles

**Affiliations:** 1 Pathogen Molecular Biology Unit, Department of Infectious and Tropical Diseases, London School of Hygiene and Tropical Medicine, London, United Kingdom; 2 Facultad de Ciencias de la Salud, Universidad de Carabobo, CNRFV-BIOMED, Sede Aragua, Maracay, Venezuela; 3 Departamento de Medicina Tropical, Instituto Oswaldo Cruz, Fiocruz, Rio de Janeiro, Brasil; Universidad de Buenos Aires, Argentina

## Abstract

**Background:**

*Rhodnius prolixus* is the main vector of Chagas disease in Venezuela. Here, domestic infestations of poor quality rural housing have persisted despite four decades of vector control. This is in contrast to the Southern Cone region of South America, where the main vector, *Triatoma infestans*, has been eliminated over large areas. The repeated colonisation of houses by silvatic populations of *R. prolixus* potentially explains the control difficulties. However, controversy surrounds the existence of silvatic *R. prolixus*: it has been suggested that all silvatic populations are in fact *Rhodnius robustus*, a related species of minor epidemiological importance. Here we investigate, by direct sequencing (mt*cytb*, D2) and by microsatellite analysis, 1) the identity of silvatic *Rhodnius* and 2) whether silvatic populations of *Rhodnius* are isolated from domestic populations.

**Methods and Findings:**

Direct sequencing confirmed the presence of *R. prolixus* in palms and that silvatic bugs can colonise houses, with house and palm specimens sharing seven *cytb* haplotypes. Additionally, mitochondrial introgression was detected between *R. robustus* and *R. prolixus*, indicating a previous hybridisation event. The use of ten polymorphic microsatellite loci revealed a lack of genetic structure between silvatic and domestic ecotopes (non-significant F_ST_ values), which is indicative of unrestricted gene flow.

**Conclusions:**

Our analyses demonstrate that silvatic *R. prolixus* presents an unquestionable threat to the control of Chagas disease in Venezuela. The design of improved control strategies is essential for successful long term control and could include modified spraying and surveillance practices, together with housing improvements.

## Author Summary

Chagas disease is spread by blood-feeding insects (triatomine bugs) that colonise poor-quality houses. Disease control relies primarily on killing domestic bugs by spraying dwellings with residual insecticide. In Venezuela, sustained control has proved difficult despite four decades of campaigns. Considered the main vector in Venezuela, the bug *Rhodnius prolixus* may also infest palm trees and might repeatedly recolonise houses from palms. A complication is that a morphologically similar species, *R. robustus*, also infests palms but is of minor medical importance. Therefore, confusion exists as to the true identity of palm bugs and their importance in disease transmission.

We applied two molecular methods (sequencing DNA of the *cytochrome b* gene, and analysing microsatellites) to triatomines collected in Venezuela so that we could identify unequivocally the species of palm-dwelling *Rhodnius* and establish their role in maintaining house infestations. We demonstrated that *R. prolixus* is indeed present in palms, and that such silvatic populations can colonise houses and are a threat to the successful control of Chagas disease in Venezuela. This finding resolves a longstanding controversy of fundamental epidemiological importance. It is also an example of the application of molecular epidemiology to correct vector identification and successful disease control.

## Introduction

Chagas disease is a chronic parasitic disease transmitted by triatomine bugs (Reduviidae: Triatominae) and limited in distribution to the Americas. The causative agent is the protozoan *Trypanosoma cruzi*. *Rhodnius prolixus* is the primary vector in Venezuela and Colombia and is one of the main targets of the Andean Pact and Central American initiatives, together with the secondary vectors *Triatoma dimidiata* in Central America, *Rhodnius pallescens* in Panama and *Rhodnius ecuadoriensis* in northern Peru [1]. In Venezuela *R. prolixus* occurs in all States, where it colonises poor quality housing and exhibits high infection rates with *T. cruzi*.

Significant progress has been made in reducing the incidence of Chagas disease in Venezuela through four decades of triatomine control [2]. Nevertheless, domestic infestations of *R. prolixus* persist and recent data indicate that transmission of *T. cruzi* may be increasing [3]. In contrast, in the Southern Cone region of South America the main vector, *Triatoma infestans,* has been eliminated over large areas following control efforts [1]. *Triatoma infestans* is considered to be a primarily domestic species, with the exception of Bolivian Andes and Gran Chaco region (Bolivia and northern Argentina) where silvatic populations were found [4]. Further studies are needed to evaluate the risk these populations pose to effective control in these regions. In comparison *R. prolixus* is reported to have a widespread silvatic distribution in Venezuela, found most commonly in palm trees and birds nests and more rarely in other sites such as dry trees [5]–[7]. The reinvasion of sprayed houses by silvatic *R. prolixus*, together with localised control failures could be maintaining disease transmission in Venezuela [3]. However, the existence of silvatic *R. prolixus* populations has been questioned due to the identification of the closely related species *Rhodnius robustus* in palm trees in Venezuela [8]. *Rhodnius robustus* poses a problem as it is virtually indistinguishable morphologically from *R. prolixus* but this species it is of minor epidemiological importance as it does not colonise houses, although flying adults may enter domestic areas attracted by light [8],[9]. Confusion has been fuelled by conflicting results of studies investigating the taxonomic status of *R. prolixus* and *R. robustus*, with morphometric and isoenzyme studies failing to detect interspecific differences [10]–[15]. However, recent DNA sequencing analyses has not only supported the validity of *R. robustus* but also indicated the existence of more than one cryptic species [16]–[18]. Additionally in a preliminary finding for this present study four *Rhodnius* specimens collected in a palm in Guarico State Venezuela were identified as *R. prolixus* [17].

Here we investigated the genetic structure of 34 populations of *R. prolixus*, including five adjacent populations, from silvatic, domestic and peridomestic ecotopes in six Venezuelan States. Our aim was to contribute to the control of Chagas disease in Venezuela, through the provision of information that might allow the design of improved control strategies. We finally resolve this controversy over the existence of silvatic *R. prolixus* and the interaction between silvatic and domestic populations. Our analyses demonstrate that silvatic *R. prolixus* presents an unquestionable threat to the control of Chagas disease in Venezuela and that successful long term control could benefit from modified spraying and surveillance practices, together with housing improvement.

## Materials and Methods

### Bug collection

**Figure 1:**
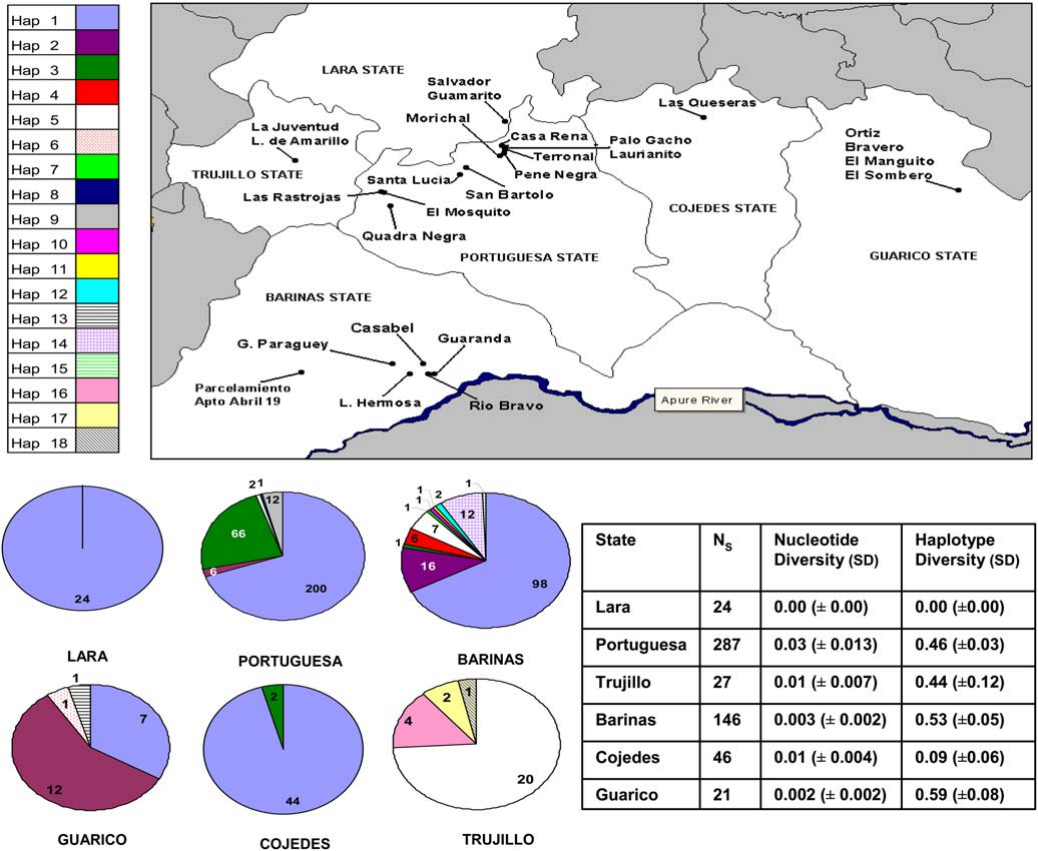
Genetic diversity (table) and haplotype distribution (pie charts) in the sampled States. The map illustrates 27 of the sampled localities in this study.

For the purpose of this study field work was carried out in 2001–2004 in the Venezuelan States of Lara, Portuguesa, Guarico, Cojedes, Barinas, and Trujillo (see Figure 1, Table 1, Table 2). Fieldwork involved the survey of palms, chicken huts and houses in localities in these States in collaboration with the Ministry of Health field inspectors.

**Table 1:** Details of the 34 populations analysed by direct sequencing.

Pop ID	State	Locality	Ecotope	Collection Date	N_S_	Haplotypes Detected	Nucleotide Diversity (SD)	Gene Diversity (SD)
Pop 1	Portuguesa	Terronal	House 1	2001	22	1,3	0.03 (±0.02)	0.46 (±0.08)
Pop 2	Portuguesa	Terronal	House 2	2001	27	1,3	0.03 (±0.02)	0.46 (±0.07)
Pop 3	Portuguesa	Terronal *	Palm 1 by pop 2	2001	30	1,2,3	0.02 (±0.01)	0.32 (±0.09)
			Palms by pop 2	2001	7			
Pop 4	Portuguesa	Terronal *	House 1	2003	10	1,3	0.037 (±0.02)	0.53 (±0.06)
			House 2	2003	4			
Pop 5	Portuguesa	Terronal	Palm 2 by pop 2	2003	35	1,3	0.02 (±0.01)	0.33 (±0.08)
Pop 6	Portuguesa	Los Rastrojos	House	2004	22	1,2	0.0004 (±0.001)	0.17 (±0.10)
Pop 7	Portuguesa	Los Rastrojos	Palm	2004	10	1	0.00 (±0.00)	0.00 (±0.00)
Pop 8	Portuguesa	Palo Gacho *	Palms	2001	10	1,3	0.04 (±0.02)	0.53 (±0.10)
Pop 9	Portuguesa	San Bartolo *	House 1 (House 25)	2002	16 (2)	1	0.00 (±0.00)	0.00 (±0.00)
			House 2 (Chicken hut)	2002	13 (2)			
Pop 10	Portuguesa	Santa Lucia *	House 89	2002	14	1,9	0.001 (±0.001)	0.44 (±0.10)
			Other houses	2002	3			
Pop 11	Portuguesa	Quebrada Negra *	Houses	2001	12	1,2,5,8	0.003 (±0.002)	0.56 (±0.15)
Pop 12	Portuguesa	Peña Negra	House	2001	10	1	0.00 (±0.00)	0.00 (±0.00)
Pop 13	Portuguesa	Casarena	House	2003	10	3	0.00 (±0.00)	0.00 (±0.00)
Pop 14	Barinas	Carreterón	Palm 1 (Palm 2)	2003	7 (1)	1	0.00 (±0.00)	0.00 (±0.00)
Pop 15	Barinas	Carreterón *	Houses	2003	13	1,3,4,5,7	0.013 (±0.008)	0.69 (±0.12)
Pop 16	Barinas	Cascabel	Chicken hut	2003	9	1,2,4	0.002 (±0.002)	0.64 (±0.13)
Pop 17	Barinas	Cascabel	House	2003	8	1,2,5	0.001 (±0.001)	0.46 (±0.20)
Pop 18	Barinas	Cascabel	Palm	2003	15	1,2,5,12	0.002 (±0.002)	0.66 (±0.08)
Pop 19	Barinas	El Guamito	House	2003	5	1	0.00 (±0.00)	0.00 (±0.00)
Pop 20	Barinas	El Guamito	Palm	2003	12	1,10,2	0.001 (±0.001)	0.32 (±0.16)
Pop 21	Barinas	Laguna Hermosa	House	2003	11	1,5	0.0004 (±0.001)	0.18 (±0.14)
Pop 22	Barinas	Laguna Hermosa	Chicken hut	2003	7	1,5,12	0.002 (±0.002)	0.52 (±0.21)
Pop 23	Barinas	Laguna Hermosa	Palm	2003	9	1,2,5,11	0.002 (±0.002)	0.58 (±0.18)
Pop 24	Barinas	G. Paguey *	House 2 (House 1)	2003	8 (3)	1,2,5	0.001 (±0.001)	0.46 (±0.20)
Pop 25	Barinas	Parcelamiento *	Palm	2003	10	1,14,15	0.001 (±0.001)	0.51 (±0.16)
Pop 26	Barinas	19 Abril	Chicken hut	2003	10	14	0.00 (±0.00)	0.00 (±0.00)
Pop 27	Barinas	Rio Bravo II	Chicken hut	2003	6	1,2	0.001 (±0.001)	0.33 (±0.22)
Pop 28	Barinas	Rio Bravo II	Palm	2003	9	1,2	0.001 (±0.001)	0.40 (±0.16)
Pop 29	Cojedes	Las Queseras	House	2004	22	1	0.00 (±0.00)	0.00 (±0.00)
Pop 30	Cojedes	Las Queseras	Palm	2004	24	1, 3	0.01 (±0.006)	0.16 (±0.10)
Pop 31	Lara	Guamito	House 1	2001	22	1	0.00 (±0.00)	0.00 (±0.00)
		Salvador	House 2	2001	2			
Pop 32	Guarico	Bravero, Ortiz	Various palms	2001	21	1,2,6,13	0.002 (±0.002)	0.59 (±0.08)
		El Manguito,						
		El Sombero						
Pop 33	Trujillo	L. de Amarillo	House	2003	21	5,16	0.003 (±0.002)	0.095 (±0.08)
Pop 34	Trujillo	La Juventud	Palm	2003	3	16,17		
		Insectary ˆ	Palm	1995	3	16,18	0.002 (±0.002)	0.73 (±0.16)
**Other**	Portuguesa	Terronal *	Chicken hut by pop2	2001	1	1	-	-
**Other**	Portuguesa	Terronal	Palms by pop 1	2003	6	1	-	-
**Other**	Portuguesa	Casarena *	Palm	2001/2003	5			
			Chicken hut	2003	2	1,3	-	-
**Other**	Portuguesa	El Mosquito *	Houses	2001	8	1,2	-	-
**Other**	Portuguesa	Palmarito *	House 1 (Palm)	2001	5 (1)	1,3	-	-
**Other**	Portuguesa	Morichal *	House 10.1 (House 10)	2001	5 (1)	1,3	-	-
**Other**	Barinas	Various ∞	House, Palm, Chicken hut	2001/3	7	1,4	-	-

**Note** N_S_ = Total no of specimens sequenced ^*^ insects from more than one sample site combined and analysed as one population, in parenthesis the no. of specimens from other populations included in the total number sequenced and the ecotope in which they originated; ˆ insectary reared bugs, originally collected in palms (source University of Los Andes, Trujillo); **Other = **specimens sequenced but not included in population analysis due to small numbers or multiple sample sites. **∞** the localities Acequita, Santa Elena de la Caramuca, Obispos and San Isidro. See Table S1, S3, and S4 for all F_ST_ values.

**Table 2:** Details of the 33 populations analysed by microsatellites.

Pop ID	State	Locality	Ecotope	Collection Date	N_M_	N_L_	L_P_	A_M_	A_R_	N_A_	H_O_	H_E_	F_IS_
Pop 1	Portuguesa	Terronal	House 1	2001	26	9	9	2.6	2.3	3	0.3	0.4	0.2
Pop 2	Portuguesa	Terronal	House 2	2001	18	9	9	2.7	2.4	1	0.4	0.5	0.1
Pop 3	Portuguesa	Terronal	Palm by pop2	2001	26	9	9	2.4	2	2	0.2	0.3	0.3
Pop 4	Portuguesa	Terronal	House 1	2003	10	9	9	2.5	2.5	2	0.4	0.5	0.1
Pop 5	Portuguesa	Terronal	Palm by pop2	2003	39	9	9	3.0	2.4	5	0.4	0.5	0.1
Pop 6	Portuguesa	Los Rastrojos	House	2004	24	10	9	2.6	2.3	1	0.3	0.4	0.03
Pop 7	Portuguesa	Los Rastrojos	Palm	2004	12	10	10	2.9	2.7	1	0.4	0.4	0
Pop 8	Portuguesa	Palo Gacho *	Palms	2001	15	9	8	2.6	2.3	-	0.3	0.4	0.2
Pop 9a	Portuguesa	San Bartolo	House 1	2002	14	9	7	2.4	2.4	1	0.4	0.4	0.1
Pop 9b	Portuguesa	San Bartolo	House 2	2002	14	9	7	2.3	2.3	3	0.3	0.4	0.3
Pop 10	Portuguesa	Santa Lucia	House 89	2002	13	9	6	1.9	1.7	-	0.2	0.3	0.1
**Pop 11**	Portuguesa	Qda Negra	Houses	2001	**NA**								
**Pop 12**	Portuguesa	Peña Negra	House	2001	**NA**								
Pop 13	Portuguesa	Casarena	House	2003	11	9	7	2.2	2.2	-	0.3	0.4	0.02
**Pop 14**	Barinas	Carreterón	Palms	2003	**NA**								
**Pop 15**	Barinas	Carreterón	Houses	2003	**NA**								
Pop 16	Barinas	Cascabel	Chicken hut	2003	11	10	10	2.8	2.7	1	0.4	0.5	0.1
Pop 17	Barinas	Cascabel	House	2003	10	10	10	3.1	3.1	2	0.5	0.6	0.1
Pop 18	Barinas	Cascabel	Palm	2003	24	10	10	4.6	3.6	1	0.5	0.6	0.1
Pop 19	Barinas	El Guamito	House	2003	11	10	10	3.3	3.2	1	0.4	0.5	0.1
Pop 20	Barinas	El Guamito	Palm	2003	20	10	10	4.0	3.3	3	0.5	0.6	0.02
Pop 21	Barinas	Laguna Hermosa	House	2003	16	10	10	3.6	3.2	1	0.6	0.6	-0.1
Pop 22	Barinas	Laguna Hermosa	Chicken hut	2003	13	10	10	2.7	2.6	-	0.4	0.5	0
Pop 23	Barinas	Laguna Hermosa	Palm	2003	17	10	10	4.3	3.6	1	0.5	0.6	0.04
Pop 24a	Barinas	G. Paguey	House 1	2003	11	10	10	3.1	3.0	-	0.4	0.6	0.2
Pop 24b	Barinas	G. Paguey	House 2	2003	12	10	10	3.4	3.2	-	0.4	0.6	0.2
Pop 24c	Barinas	G. Paguey	Palm 1	2003	12	10	10	3.4	3.2	-	0.5	0.6	0.1
Pop 24d	Barinas	G. Paguey	Palm 2	2003	11	10	10	3.4	3.3	-	0.5	0.6	0.01
Pop 25	Barinas	Parcelamiento*	Palms	2003	13	10	10	3.5	3.2	4	0.5	0.6	0.1
Pop 26	Barinas	19 Abril	Chicken hut	2003	13	10	10	2.7	2.5	-	0.4	0.5	0.1
Pop 27	Barinas	Rio Bravo II	Chicken hut	-	17	10	10	3.0	2.7	3	0.5	0.5	0.01
Pop 28	Barinas	Rio Bravo II	Palm	-	10	10	10	3.6	3.5	2	0.5	0.6	0.1
Pop 29	Cojedes	Las Queseras	Palm	2004	24	10	10	3.0	2.6	3	0.4	0.5	0.1
Pop 30	Cojedes	Las Queseras	House	2004	24	10	10	2.2	2.1	1	0.3	0.4	0.2
Pop 31	Lara	Guamito	House 1	2001	15	9	9	2.2	2.0	1	0.3	0.4	0.2
		Salvador	House 2		2								
**Pop 32**	Guarico	Brav., Ortiz	Various palms	2001	**NA**								
		El Man. El Som.											
Pop 33	Trujillo	Loma de Amarillo	House	2003	26	9	9	3.0	2.2	3	0.2	0.3	0.3
**Pop 34**	Trujillo	La Juv./Insectary	Palm	2003/1995	**NA**								
Pop 35	Portuguesa	Laurianito	Chicken hut	2003	21	9	9	3.1	2.7	-	0.4	0.5	0.1

**Note** ^*^ insects from more than one sample site combined and analysed as one population; N_M_ = Total no. of specimens analysed by microsatellites; N_L_ = no of loci analysed; L_p_ = no. of polymorphic loci; A_M = _mean no of alleles detected averaged over all loci, A_R_ = allele richness averaged over all loci; N_A_ = Null alleles; H_O_, H_E_ = Observed and Expected Heterozygosity averaged over all loci; F_IS = _Inbreeding Coefficient averaged over all loci. **NA** = population not analysed by microsatellites. See Tables S1, S3, and S4 for all F_ST_ values.

### Sampling methods

Silvatic collections were made with Noireau live bait traps [19]. Palm dissection was also used with the consent of landowners. The palm was cut at the base and cleared from the base up to the crown using a machete, removing and inspecting each layer. Domestic and peridomestic collections were made by the traditional search and capture method, with prior consent of householders. All bugs collected were placed in collection tubes, noting date and place of collection. Specimens were identified using the keys of Lent and Wygodzinsky (1979) [20].

#### In Portuguesa State

Bugs were collected in 12 localities from houses, chicken huts and palms. Positive houses were primarily of the traditional ‘rancho’ type, constructed of wattle and daub with palm and corrugated iron roofs. A total of 287 specimens were analysed by direct sequencing and 243 by microsatellite analysis (pop 1 through pop 13 and pop 35; see Table 1, Table 2 for population details).

#### In Barinas State

Bugs were collected in 13 localities from houses, chicken huts and palms. In these localities houses had walls of wood or cement blocks, with metal or palm roofs. A total of 146 specimens from domestic, silvatic and peridomestic ecotopes in this State were analysed by direct sequencing and 221 by microsatellite analysis (pop 14 through pop 28; see Table 1, Table 2).

#### In Cojedes State

A single house infestation was detected in the locality Las Queseras. A dissected palm adjacent to the infested house was also positive. A total of 46 specimens were analysed by direct sequencing and 48 by microsatellites (pop 29, pop 30; see Table 1, Table 2).

#### In Lara State

Two houses were found infested in the localities Guamarito and Salvador, while palm searches proved negative. A total of 24 specimens from this State were examined by direct sequencing, 17 by microsatellite analysis (pop 31; see Table 1, Table 2).

#### In Guarico State

Specimens were collected in 4 localities (El Sombero, El Manguito, Bravero, Ortiz). All houses inspected were negative and samples were isolated from palms only. In these areas the traditional rancho was replaced by cement block structures as part of the National Programme for housing improvement in the 1960s. A total of 21 specimens were analysed by direct sequencing only (pop 32; see Table 1).

#### In Trujillo State

A single house was found infested in the locality Loma de Amarillo. A single palm was dissected in the locality La Juventud and was found positive. A total of 27 specimens were analysed by direct sequencing, including 3 insectary specimens derived from palms. Twenty-six domestic specimens were analysed with microsatellites (pop 33, 34; see Table 1, Table 2).

Genomic DNA was isolated from specimens using Qiagen Dneasy extraction kit following the manufacturer's protocol for isolation of DNA from animal tissues.

### Species identity and genetic relatedness

#### *Cytb* sequencing

**Figure 2:**
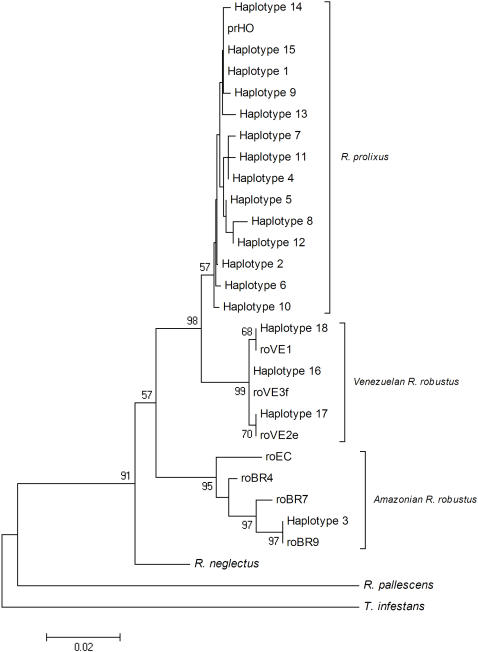
Phylogenetic tree of the 18 *cytb* haplotypes detected in the study and sequences from GenBank.

**Figure 3:**
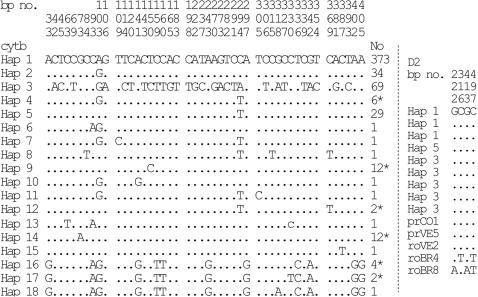
The polymorphic sites of the 18 *cytb* haplotypes and 9 specimens sequenced for D2.

In order to confirm which species of *Rhodnius* were present and to examine the genetic relatedness of *R. prolixus* and *R. robustus* populations in Venezuela, a total of 551 specimens were analysed from 6 States by direct sequencing of a fragment of the mitochondrial gene cytochrome b (*cytb)* (Table 1). Eight published *cytb* nucleotide sequences from the study of Monteiro *et al.* 2003 were included as reference specimens (see Figure 2) [17]. In Monteiro *et al.* 2003 specimens of *R. robustus* and *R. prolixus* were distinguished using a combination of the following criteria (1) the morphology of late nymphal stages, as described by Lent and Wygodsinsky (1979, [20]) (2) by the inclusion in *cytb* typing of *R. robustus* specimens originally collected from areas close to the suggested ‘type localities’ of the species and specimens from the Brazilian Amazon where silvatic *R. prolixus* is not believed to occur (3) and by the inclusion of *R. prolixus* specimens collected from houses in Central America (Honduras/Guatamala), beyond the geographical distribution of silvatic *R. prolixus* or *R. robustus* [17]. To test for mtDNA introgression between these closely related species, a fragment of the D2 variable region of 28S RNA was sequenced for nine specimens, characterised by the mt*cytb* analysis as *R. robustus* or *R. prolixus*. Five D2 sequences were also available in GenBank (see Figure 3).

We amplified a 682 bp fragment of the *cytb* gene and a 633 bp fragment of the D2 region with the following primers: Forward *cytb*7432F 5′-GGACG(AT)GG(AT)ATTTATTATGGATC; Reverse *cytb*7433R 5′-GC(AT)CCAATTCA(AG)GTTA(AG)TAA; Forward D2F 5′-GCGAGTCGTGTTGCTTGATAGTGCAG; Reverse D2R 5′-TTGGTCCGTGTTTCAAGACGGG [17],[21]. Reaction conditions were: *cytb*: 95°C 5 min; 35 cycles of 95°C 30 s, 50°C 45 s, and 72°C 45 s; final extension of 72°C for 5 min. D2: 25 cycles 94°C 1 min, 50°C 2 min, 72°C 2 min. Amplicons were purified using Qiaquick kit (Qiagen) or Quick-clean (Bioline), as specified by the manufacturers. Purified PCR products were sequenced by fluorescent dye terminator chemistry using ABI Prism Bigdye (Applied Biosystematics), on an ABI Prism 377 automated DNA sequencer (PE Applied Biosystematics) or on a 48 capillary ABI 3730 DNA analyser. Forward and reverse sequences were aligned using Sequence Navigator V1.01 (Perkin-Elmer) or BioEdit V7.0.4.1 [22] and a consensus sequence produced. Sequence identity was confirmed by comparison with data in GenBank.

The number of variable sites was determined using Mega v 2.1 software [23]. A neighbour-joining tree was created in Mega v 2.1 using the Kimura-2 parameter model of sequence evolution [23]. Statistical support for clades was assessed by the bootstrap method (1000 replications; [24]). Outgroup sequences were taken from GenBank: *R. pallescens* AF045720, *R. neglectus* AF045716 and *T. infestans* AF045721. All sites were equally weighted.

#### Data deposition footnote

*Cytb* haplotype genBank accession numbers. EF043576, EF043577, EF043578, EF043579, EF043580, EF043581, EF043582, EF043583, EF043584, EF043585, EF043586, EF043587, EF043588.

### Genetic variation and population structure

#### *Cytb* analysis

For population analysis using *cytb* haplotypes specimens were placed into 34 population groups as listed in Table 1. Groups were determined by the collection site (ecotope). Ideally population groups consisted of specimens isolated from a single ecotope, however when only a few specimens were collected, populations from different houses or palms from the same locality and State were combined (Table 1). Intrapopulation population comparisons, was investigated using the index of population heterogeneity F_ST_ (Weir & Cockerhams 1984 unbiased estimator) generated in Arlequin v3.1 [25],[26]. The F_ST_ null distribution is obtained by permuting the haplotypes between the compared populations (10,000 times), given a null hypothesis of no difference between the populations (F_ST_ = 0). The p-value generated is the fraction of these permutations with an F_ST_ larger than or equal to the original estimate, if the given p-value is smaller than the nominated significance level, then the compared populations are considered to be significantly different. The nominal significance level was adjusted for multiple comparisons using the sequential Bonferroni procedure [27]. This consists of setting a lower threshold for the nominal significance level, i.e., for *cytb* analysis k = 561, p1 = 0.05/561 and p≤0.0001. Population geneflow was evaluated at different geographic levels 1) comparison of adjacent ecotopes, 2) comparison of populations within localities, 3) comparison of populations within and between States.

The genetic divergence of the populations was also estimated by an analysis of molecular variance (AMOVA see Table 3) using Arlequin v3.1 [25],[28]. Genetic divergence was based on pairwise differences between haplotypes and structure was evaluated at different geographic levels as above. Total genetic variance was partitioned into variation due to the differences between individuals within populations (within population polymorphism) and that caused by the differences among populations (among population polymorphism). Pairwise differences between haplotypes were used to calculate related F statistic analogues, while significance levels for these indices (p = 0.05) were calculated by non-parametric permutation (10,000).

**Table 3:** Results of hierarchical analysis (AMOVA) of population.

Structure	Populations within Group	F_ST_	Among Populations%	Within Populations%	p-Value *
**Adjacent populations**					
*cytb*					
1	pop 2, pop 3	0.05	4.7	95.3	0.14
1	pop 2, pop 5	0.01	1.3	98.7	0.25
1	pop 3, pop 5	−0.02	−2.4	102.4	0.76
1	pop 29, pop 30	0.04	3.83	96.1	0.49
1	pop 6, pop 7	−0.01	−0.46	100.46	0.56
1	pop 27, pop 28	−0.15	−15.01	115.01	1.0
microsatellite					
1	pop 2, pop 3	0.2	20.0	80.0	0.00
1	pop 2, pop 5	0.003	0.3	99.7	0.39
1	pop 3, pop 5	0.17	17.1	82.9	0.00
1	pop 29, pop 30	0.15	14.7	85.3	0.00
1	pop 6, pop 7	0.04	4.5	95.5	0.01
1	pop 27, pop 28	0.03	3.4	96.6	0.07
**Within localities**					
***cytb***					
1	pop 14, pop 15	0.03	2.7	97.3	0.39
1	pop 16, pop 17, pop 18	−0.03	−2.7	102.7	0.55
1	pop 19, pop 20	−0.04	−4.26	104.3	0.08
1	pop 21, pop 22, pop 23	0.01	1.07	98.9	0.35
microsatellite					
1	pop 9a, pop 9b	−0.02	−2.4	102.4	0.99
1	pop 16, pop 17, pop 18	0.02	2.4	97.6	0.05
1	pop 16, pop 17	0.04	4.4	95.6	0.08
1	pop 16, pop 18	0.04	4.2	95.8	0.01
1	pop 17, pop 18	−0.004	−0.4	100.4	0.64
1	pop 19, pop 20	−0.01	−0.8	100.8	0.77
1	pop 21, pop 22, pop 23	0.03	3.1	96.9	0.003
1	pop 21, pop 22	0.06	5.8	94.2	0.001
1	pop 21, pop 23	0.004	0.4	99.6	0.3
1	pop 22, pop 23	0.02	2.5	97.5	0.05
1	pop 24a, 24b, 24c, 24d	0.02	2.0	98.0	0.07
**Within States**					
*cytb*					
1	pop 1–pop 13	0.38	38.9	61.1	0.00
1	pop 14–pop 28	0.15	14.9	85.1	0.00
microsatellite					
1	pop 1–10, pop 13, pop 35	0.11	11.4	88.6	0.00
1	pop 16– pop 28	0.03	3.3	96.7	0.00
**Among States**					
*cytb*					
1	Portuguesa, Barinas, Lara Cojedes, Trujillo, Guarico	0.15	15.5	84.5	0.00
microsatellite					
1	Portuguesa, Barinas, Cojedes, Trujillo, Lara	0.07	7.3	92.7	0.00
**All populations**					
*cytb*					
1	All 34	0.44	43.61	56.39	0.00
microsatellites					
1	All 33	0.11	11.3	88.7	0.00

Note: p-value corresponds to the probability of obtaining random values larger or equal than the observed value.

#### Microsatellite analysis

A total of 555 *R. prolixus* specimens, from silvatic, domestic and peridomestic ecotopes in five States were used for microsatellite amplification. Specimens were grouped into 33 populations determined by the collection site (ecotope) as listed in Table 2. Specimens were analysed at a total of 9 microsatellite loci, and at a 10^th^ locus for a subset of 20 populations (Table 2). The 10 primers, flanking dinucleotide repeats, were isolated and amplified as described elsewhere [29]. Linkage disequilibrium was tested between all pairs of loci in each population using the program GENEPOP version 3.4 [30]. These results will be reported elsewhere (Fitzpatrick *et al* in preparation) but in brief significant linkage disequilibrium was detected between three loci pairs after Bonferroni correction in three populations; LIST14-017 and LIST14-042 in pop 9a, LIST14-010 and LIST14-013 in pop 20, LIST14-010 and LIST14-025 in pop 29 (Table 2). As these microsatellite loci did not exhibit significant linkage in each of the 33 population analysed, they were determined to be in linkage equilibrium. Observed (H_O_) and expected heterozygosity (H_E_) were calculated for each locus using the program Arelquin V2.000 [25]. Allele richness was calculated using FSTAT version 2.932 [31]. Deviation from Hardy-Weinberg equilibrium (HWE) was tested at each locus within each individual populations using a modified Markov chain randomisation method of Guo and Thompson (1992) (Arlequin V3.1, 10,000 steps; [25],[32]). Wright's inbreeding coefficient F_IS_ was also calculated at each locus following Weir and Cockerham (1984) (GENEPOP version 3.4; [26],[30]). Genetic diversity in each population was measured in four ways: (i) Expected heterozygosity (He); (ii) mean number of alleles (iii) allele richness; and (iv) polymorphic loci.

Intrapopulation comparisons were based on the indices of population homogeneity F_ST_ (Weir & Cockerham's 1984)_,_ as previously detailed [25],[26]. Nominal significance level was adjusted, as previously with k = 528, p1 = 0.05/528, p≤0.0001 for 9 loci and k = 190, p1 = 0.05/190, p≤0.0003 for 10 loci [27]. Population geneflow was evaluated at different geographic levels 1) comparison of adjacent ecotopes, 2) comparison of populations within localities, 3) comparison of populations within and between States.

The genetic divergence of the populations was also estimated by an analysis of molecular variance (AMOVA) using Arlequin v3.1 [25],[28]. Genetic divergence was based on the number of different alleles detected (F_ST_-like) and populations evaluated at different geographic levels as above (Table 3). The total genetic variance was partitioned into variation due to the differences between individuals within populations (within population polymorphism) and that caused by the differences among populations (among population polymorphism). Significance levels (p = 0.05) for the F statistic analogues were calculated by non-parametric permutation (10,000).

The relationship between geographical and genetic distance over the study area was assessed by testing the correlation between F_ST_/(1−F_ST_) and log transformed (ln) geographic distances. Rousset (1997) showed that a linear relationship occurs between natural log of geographical distance and F_ST_/(1−F_ST_) in two dimensional habitats [33]. The significance of the correlation was examined by a Mantel test using a permutation procedure (9,999 permutations) in GenAlex [34].

## Results

### Species identity and genetic relatedness

#### *Cytb* haplotypes

**Figure 4:**
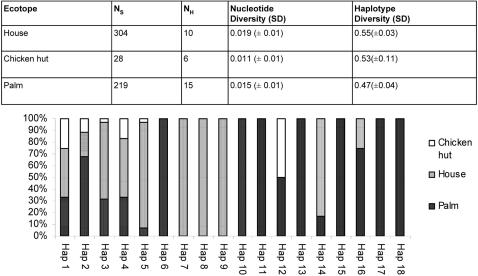
Genetic diversity and haplotype distribution in the sampled ecotopes.

A total of 551 specimens were analysed from six States by direct sequencing of a fragment of the mitochondrial gene *cytochrome b* (*cytb*). This included 304 specimens from houses, 219 from palms and 28 from chicken huts (Figure 4). A 415 bp consensus sequence was produced for 541 specimens (Figure 3); and a slightly shorter consensus (392–408 bp) for 10 specimens. There were 18 *cytb* haplotypes; 14 of which were unique to single States and eight occurred once (Figure 1). The haplotypes varied at 46 sites (11.1% polymorphism). All variable sites were point mutations; 16 sites were parsimony informative (3.9%). Haplotype frequencies varied by State (Figure 1). A single haplotype was detected in Lara (haplotype 1), whereas 11 haplotypes were found in Barinas, including seven unique to that State; in Portuguesa State haplotypes 1 (67%) and 3 (33%) were dominant. Overall, haplotype 1 was the most common haplotype in the study (68% of specimens) and was present in all States, apart from Trujillo. Nucleotide diversity was highest in Portuguesa State, and lowest in Lara State, while haplotype diversity was highest in Guarico State, and lowest in Lara State.

#### Species identity

In comparison with published sequences in GenBank our 18 haplotypes shared greatest similarity with *R. prolixus* (14 haplotypes) and *R. robustus cytb* sequences (4 haplotypes). Identity scores for haplotypes 1, 2, 4–15 were highest for a *R. prolixus* specimen from Honduras (99–100%; prHo AF421339) whilst haplotype 3 and haplotypes 16–18 were most similar to *R. robustus* from the Amazon (98–100%; roBR7 AF421343) and Venezuela (roVE1 AF421340). The most common haplotype in the study (haplotype 1) was identical to *R. prolixus* from Honduras (prHO AF421339).

#### The identity of silvatic *Rhodnius*

Silvatic and domestic specimens were collected in each State, with the exception of Lara, where palms were negative and Guarico, where houses were negative. Haplotype distribution varied by ecotope with seven haplotypes found exclusively in palms and three exclusively in houses (Figure 4). Nucleotide and haplotype diversity was similar in both houses and palms (Figure 4). Significantly 11 of the *R. prolixus* haplotypes were identified in palms, thus confirming the existence of silvatic *R. prolixus*. In addition five *R. prolixus* haplotypes were common to both palms and houses (Figure 4). Importantly, both nymphs and adults were detected in houses for the shared haplotypes 1, 2 and 5 (109 nymphs in total) indicating that silvatic *R. prolixus* is capable of domestic colonisation.

However, silvatic *R. robustus* was also identified in this study in the Andean state of Trujillo. Specimens from a palm dissected in the locality La Juventud were determined as Venezuelan *R. robustus* (haplotypes 16–18) (pop 34). In a previous study in this area adult *Rhodnius* specimens, thought to be *R. robustus*, were found to enter houses at night to feed, attracted by light but not to colonise [35]. In our study a single *R. robustus* adult (haplotype 16) was found in an infested house in the Locality Loma de Amarillo (pop 33), however all of the other domestic specimens were *R. prolixus* (haplotype 5), including all nymphs, thus indicating that this *R. robustus* adult may also have arrived to feed but had not colonised the house.

#### Mitochondrial introgression

The *R. robustus* haplotype 3 was the second most frequent haplotype (13%), although this was limited in distribution to Portuguesa State, with the exception of three specimens. As mentioned the species *R. robustus* is not known to colonise houses, we were therefore surprised to find 14 nymphs of *R. robustus* haplotype 3 in four houses in the localities Terronal, Casarena and Palmarito thus suggesting that this silvatic species is capable of domestic colonisation in this Venezuelan State (see Table 1; pop 1, pop 2, pop 13, other). Accordingly, we investigated mitochondrial introgression between *R. robustus* haplotype 3 and *R. prolixus* by sequencing a fragment of the nuclear target D2. The D2 sequence alignments (519 bp) revealed three haplotypes, varying at four sites (Figure 3). Strikingly, *R. robustus* (haplotype 3) had a D2 haplotype that was identical to *R. prolixus* (haplotype 1, 5) (Figure 3), while Amazonian *R. robustus* from GenBank, roBR4 and roBR8, presented two different D2 haplotypes. Thus indicating an introgression event, and that the 14 nymphs above were *R. prolixus* with introgressed *R. robustus* mitochondrial haplotype 3.

#### Genetic relatedness and phylogenetics

From the alignment of the polymorphic sections of the 18 haplotypes it is clear that *R. robustus* haplotype 3 and haplotypes 16–18 are the most divergent (genetic distance 0.07–0.09 and 0.03–0.09 respectively, Kimura-2 parameter Figure 3). While *R. prolixus* haplotypes 1, 2 and haplotypes 4–15 were very similar, separated by only 1–4 base pair changes (genetic distance 0.002–0.015; Kimura-2 parameter, Figure 3).

In our phylogenetic tree the 18 haplotypes divided into two major clades with high bootstrap values (Figure 2, clades I and II). Within clade I two main groups were visible, 1) *R. prolixus* haplotypes 1, 2, 4–15 and prHO and 2) Venezuelan *R. robustus* haplotypes 16–18, roVE1, roVE2e and roVE3f (99% bootstrap support). While clade II is composed of Amazonian *R. robustus* haplotypes. Within this group, haplotype 3 was identical to roBR9 (*R. robustus* from the Brazilian Amazon). These results indicate a closer genetic relationship between Venezuelan *R. robustus* and *R. prolixus* than *R. robustus* from the Amazon region giving further support to the existence of cryptic species within *R. robustus* [17].

### Genetic variation and population structure

Our specific interest, in the context of detecting movement between silvatic and domestic *Rhodnius* populations, was to genotype adjacent silvatic and domestic populations, before examining the relationship between more geographically distant populations.

#### Mitochondrial DNA and population structure

For population analysis using *cytb* haplotypes specimens were placed into 34 population groups as listed in Table 1.

##### Comparisons between adjacent ecotopes

To test for possible geneflow between silvatic, domestic and peridomestic areas, comparisons were made initially between five population pairs in adjacent ecotopes. (Table 4, see Table S1 for all F_ST_ values). Pairwise F_ST_ values indicated a lack of population division between four adjacent house and palm populations; 1) pop 29 and pop 30 ( F_ST_ = 0.04, p = 0.49); 2) pop 6 and pop 7 (F_ST_ = −0.005, p = 0.55); 3) pop 2 and pop 5 (F_ST_ = 0.01, p = 0.26) and 4) pop 2 and pop 3 (F_ST_ = 0.05, p = 0.14) and between adjacent palm and chicken hut ecotopes, pop 27 and pop 28 (F_ST_ = −0.15, p = 1.0). Additionally the two palms adjacent to pop 2, but sampled in different years, were not genetically different (pop 3 and pop 5; F_ST_ = −0.02, p = 0.77). These *cytb* results indicated that bugs can move between silvatic, peridomestic and domestic habitats, thus indicating geneflow. The divergence of adjacent populations was also estimated by AMOVA (see Table 3). The amount of variation due to within population polymorphism was greater than between populations indicating that no heterogeneity is present and suggesting a lack of population structure between ecotopes (F_ST_ range = −0.02 to 0.05).

**Table 4:** Summary of geneflow.

Population	State	Locality	Compared Ecotope	N_L_	H_S_	F_ST_ Values **	
						*Cytb*	Microsatellites
Pop 29,Pop 30 *	Cojedes	Las Queseras	Palm, House	9	1	Yes, F_ST_ = 0.04 p = 0.49	No, F_ST_ = 0.15 p = <0.0001
Pop 27,Pop 28 *	Barinas	Rio Bravo II	Chicken hut, Palm	10	1, 2	Yes, F_ST_ = −0.15 p = 1.0	Yes, F_ST_ = 0.04 p = 0.045
Pop 6,Pop 7*	Portuguesa	Los Rastrojos	House, Palm	10	1	Yes, F_ST_ = −0.005 p = 0.55	Yes, F_ST_ = 0.04 p = 0.007
Pop 2,Pop 3 *	Portuguesa	Terronal	House, Palm	9	1, 3	Yes, F_ST_ = 0.05 p = 0.19	No, F_ST_ = 0.2 p = <0.0001
Pop 2,Pop 5 *	Portuguesa	Terronal	House, Palm	9	1, 3	Yes, F_ST_ = 0.01 p = 0.26	Yes, F_ST_ = 0.002 p = 0.43
Pop 3,Pop 5*	Portuguesa	Terronal	Palm, Palm	9	1, 3	Yes, F_ST_ = −0.02 p = 0.77	No, F_ST_ = 0.17 p = <0.0001
Pop 1,Pop 4	Portuguesa	Terronal	House, House	9	1, 3	Yes, F_ST_ = −0.03 p = 0.71	Yes, F_ST_ = 0.046 p = 0.023
Pop 1,Pop 2	Portuguesa	Terronal	House, House	9	1, 3	Yes, F_ST_ = 0.18, p = 0.02	Yes, F_ST_ = 0.032 p = 0.02
Pop 4,Pop 3	Portuguesa	Terronal	House. Palm	9	1, 3	Yes, F_ST_ = 0.30, p = 0.006	Yes, F_ST_ = 0.061 p = 0.023
Pop 4,Pop 5	Portuguesa	Terronal	House, Palm	9	1, 3	Yes, F_ST_ = 0.24 p = 0.015	Yes, F_ST_ = 0.071 p = 0.0015
Pop 9a,Pop 9b	Portuguesa	San Bartolo	House	9	1	-	Yes, F_ST_ = −0.023 p = 0.99
Pop 17,Pop 18	Barinas	Cascabel	House, Palm	10	1,2,5	Yes, F_ST_ = 0.009 p = 0.37	Yes, F_ST_ = 0.004 p = 0.40
Pop 16,Pop 17	Barinas	Cascabel	House, Chicken hut	10	1,2	Yes, F_ST_ = 0.038 p = 0.29	Yes, F_ST_ = 0.034 p = 0.105
Pop 16,Pop 18	Barinas	Cascabel	Chicken hut, Palm	10	1,2	Yes, F_ST_ = −0.08 p = 0.99	Yes, F_ST_ = 0.047 p = 0.006
Pop 19,Pop 20	Barinas	El Guamito	House, Palm	10	1	Yes, F_ST_ = −0.04 p = 0.77	Yes, F_ST_ = −0.008 p = 0.74
Pop 21,Pop 23	Barinas	L. Hermosa	House, Palm	10	1,5	Yes, F_ST_ = 0.04 p = 0.32	Yes, F_ST_ = 0.001 p = 0.38
Pop 21,Pop 22	Barinas	L. Hermosa	House, Chicken hut	10	1,5	Yes, F_ST_ = 0.03 p = 0.43	Yes, F_ST_ = 0.06 p = 0.0006
Pop 23,Pop 22	Barinas	L. Hermosa	Palm, Chicken hut	10	1,5	Yes, F_ST_ = −0.03 p = 0.59	Yes, F_ST_ = 0.034 p = 0.013
Pop 24a,Pop 24c	Barinas	G. Paguey	House, Palm	10	-	-	Yes, F_ST_ = 0.026 p = 0.145
Pop 24a,Pop 24d	Barinas	G. Paguey	House, Palm	10	-	-	Yes, F_ST_ = 0.032 p = 0.078
Pop 24b,Pop 24c	Barinas	G. Paguey	House, Palm	10	-	-	Yes, F_ST_ = 0.005 p = 0.483
Pop 24b,Pop 24d	Barinas	G. Paguey	House, Palm	10	-	-	Yes, F_ST_ = 0.017 p = 0.19
Pop 24a,Pop 24b	Barinas	G. Paguey	House, House	10	1	-	Yes, F_ST_ = 0.018 p = 0.279
Pop 24c,Pop 24d	Barinas	G. Paguey	Palm, Palm	10	-	-	Yes, F_ST_ = 0.021 p = 0.132

*: Adjacent populations, N_L_ = no of loci amplified, H_S_ = shared haplotypes.

**: Sequential Bonferroni correction applied to F_ST_ p values (For cytb k = 561, p1 = 0.05/561, p≤0.0001; Microsatellite: for 9 loci k = 528, p1 = 0.05/528 p≤0.0001, for 10 loci k = 190, p1 = 0.05/190 p≤0.0003).; yes = geneflow, no = no geneflow, -population not sequenced, see Table 1, Table 2 and S1, S3, S4 for all population comparisons F_ST_ values.

##### Comparisons within localities

To detect possible geneflow between more geographically distant populations, non adjacent populations within individual localities were compared (Table 3, Table 4, see Table S1 for all F_ST_ values). Pairwise F_ST_ values indicated a lack of population division between a palm and house sampled within the locality El Guamito (pop 19 and pop 20; F_ST_ = −0.04, p = 0.78), between a house, chicken hut and palm in the locality Cascabel (pop 16, 17 and 18; F_ST_ range = −0.08 to 0.04, p = 0.29 to 0.99), also within the locality Laguna Hermosa (pop 21, 22 and 23; F_ST_ range = −0.03 to 0.04, p = 0.32 to 0.6) and between houses in the locality Terronal (pop 1 and pop 4; F_ST_ = −0.03, p = 0.72). The population divergence within localities was also estimated by AMOVA (see Table 3). Again the variation due to within population polymorphism was greater than between population polymorphism in individual localities indicating a lack of structure (F_ST_ range = −0.04 to 0.03) (Table 3).

##### Comparison within States

###### Portuguesa State

A total of 287 specimens from domestic, silvatic and peridomestic ecotopes in this State were analysed by direct sequencing. For population analysis specimens were divided into 13 populations (Table 1). A hierarchical analysis of all populations within Portuguesa detected a greater within population diversity (61%) than between population diversity (39%), however F_ST_ indicated structure does exist between populations in this State (F_ST_ = 0.38, p = 0, Table 3). Detected heterogeneity in this State was primarily related to domestic populations from Santa Lucia (pop 10), Casarena (pop 13), and palm population (pop 1) (F_ST_ range = 0.12 to 1.0, pairwise F_ST_). When populations in Portuguesa were further analysed in ecotope groupings variation was greatest within populations (64%) in comparison to among groups (−9.2) or among populations within groups (45%) (F_ST_ = 0.41 p = 0, F_SC_ = 0.36 p = 0, F_CT_ = −0.1 p = 0.7; AMOVA 2 groups, house <pop 1,2,4,6, 9,10,11,12,13> palm <3,5,7,8>).

###### Barinas State

A total of 146 specimens from domestic, silvatic and peridomestic ecotopes in this State were analysed by direct sequencing. For population analysis specimens were divided into 15 groups (Table 1). A hierarchical analysis of all populations within Barinas detected a greater within population diversity (85%) than between populations (15%), however F_ST_ indicated that structure does exist between populations in this State (F_ST_ = 0.15, p = 0, (Table 3). In population comparisons (pairwise F_ST_) detected heterogeneity was due to a peridomestic population (pop 26), which was different from the majority of populations in Barinas (F_ST_ range = 0.28 to 1.0). When populations in Barinas were further analysed in ecotope groupings variation was greatest within populations (85%) in comparison to among groups (1.2%) or among populations within groups (14%) (F_ST_ = 0.15 p = 0, F_SC_ = 0.14 p = 0, F_CT_ = 0.001 p = 0; AMOVA 3 groups, house <15,17,19,21,24> palm <14,18,20,23,25,28> chicken hut <16,22,26,27>).

###### Trujillo State

A total of 27 specimens of domestic (pop 33) and silvatic origin (pop 34) were analysed by direct sequencing. Gene flow not evident between these two ecotopes (F_ST_ = 0.91, p<0.0001).

##### Comparison between States

A hierarchical analysis of all 34 populations analysed by *cytb* revealed population structure, with similar level of polymorphism detected within (56%) and between populations (44%) (F_ST_ = 0.44, p = 0, Table 3). When specimens were analysed further by their State of collection (1 group: Portuguesa, Barinas, Guarico, Cojedes, Trujillo, Lara) genetic isolation was detected (F_ST_ = 0.15 p = 0), however variation was greater within individual States than between States (15.5%) (Table 3). Additional hierarchal analysis between State populations was carried out (5 groups; Portuguesa <pop 1–13>, Barinas <pop 14–28> Cojedes <pop 29, 30>, Trujillo <pop 33, 34> Other <pop 31, 32>). Again variation was greatest within populations (55%) in comparison to among groups (8%) or among populations within groups (37%) (F_ST_ = 0.45 p = 0, F_SC_ = 0.4 p = 0, F_CT_ = 0.08 p = 0.1).

#### Microsatellite analysis and population structure

In parallel with mitochondrial analyses, population structures, in particular for adjacent domestic and silvatic populations, were re-examined using high resolution microsatellites. A total of 33 populations were analysed (Table 2). The number of polymorphic loci in populations ranged from 6–10, with 85% of all populations polymorphic at all loci (Table 2). Monomorphic loci were detected in a number of populations, ranging from three loci in pop 10 (Santa Lucia) to one locus in pop 8 (Palo Gacho) (Table 2). The allele richness per population varied from 1.7 (pop 10) to 3.6 (pop 18) (Table 2). The number of private alleles detected in the study was low, nine in total, four of which occurred in a single domestic population in Loma de Amarillo, Trujillo State (pop 33). Mean observed heterozygosity ranged from 0.2 to 0.6 and expected heterozygosity between 0.3 to 0.6 (Table 2). Loci in each population were tested for significant departure from Hardy-Weinberg equilibrium (HWE); six loci in 17 populations were significant after sequential Bonferroni correction (see Tables S2a, S2b). Departures were primarily related to excess homozygosity at locus LIST14-017 (12 populations). F_ST_ values generated including and excluding List14-017 were significantly correlated [Mantel test R2 = 0.9, p<0.001, 34] and this locus was therefore included in the analysis. Departures from HWE were also related to excess heterozygosity at locus LIST14-013 (1 population) and LIST14-056 (2 populations). Null alleles can be problematical in microsatellite analysis and can result in departures from HWE. Here 54 specimens consistently failed to amplify at single locus and 2 specimens at two loci in 23 populations (Table 2).

##### Comparisons between adjacent ecotopes

Geneflow between our five adjacent ecotope pairs was re-examined using microsatellite analysis (Table 2, Table 3, see Table S3 and Table S4 for all F_ST_ values). Pairwise F_ST_ comparisons indicated a lack of population structure between three of the adjacent ecotopes; 1) between a house and palm (pop 2 and pop 5; F_ST_ = 0.002 p = 0.43), 2) pop 6 and pop 7 (F_ST_ = 0.04 p = 0.007) and 3) between a palm and chicken hut (pop 27 and pop 28; F_ST_ = 0.04 p = 0.045). These results reaffirm that bugs move between silvatic, peridomestic and domestic ecotopes. However, further heterogeneity was uncovered by microsatellite analysis between the remaining adjacent populations, in contrast to *cytb* analysis; between a palm and house (pop 29 and pop 30; F_ST_ = 0.15 p<0.0001) and pop 2 and pop 3 (F_ST_ = 0.2 p<0.0001), also between two palm populations (pop 3 and pop 5; F_ST_ = 0.17 p<0.0001).

The divergence of adjacent populations was also estimated by AMOVA (see Table 3). The amount of variation due to within population polymorphism was greater than between populations and geneflow was also confirmed, with the exception of pop 6 and pop 7 (house and palm). F_ST_ comparisons were significant in the absence of bonferroni correction (F_ST_ = 0.04, p = 0.01).

##### Comparisons within localities

Geographically distant populations from non adjacent ecotopes within individual localities were also re-examined. For example using microsatellite data panmixia was detected between a house, palm and chicken hut population within the locality Laguna Hermosa (pop 21, 22, 23; F_ST_ range = 0.002 to 0.06, p = 0.0006 to 0.38), also within the locality Cascabel (pop 16, 17, 18; F_ST_ range = 0.004 to 0.05, p = 0.006 to 0.40). In the locality El Guamito a single house and palm were homogenous (pop 19, pop 20; F_ST_ = −0.008, p = 0.74). F_ST_ comparisons detected population homogeneity within the locality G. Paguey between two house and two palm populations (pop 24a,24b,24c,24d; F_ST_ range = 0.005 to 0.03, p = 0.14 to 0.48). These results agreed with *cytb* analysis. Also panmixia was also evident within the locality Terronal; between houses (pop 1, pop 2; F_ST_ = 0.03, p = 0.02), between a house and palm (pop 3, pop 4; F_ST_ = 0.06 p = 0.02) and between populations collected from the same house in different years (pop 1, pop 4; F_ST_ = 0.05, p = 0.02). In the locality San Bartolo, no genetic structure was detected between two houses (pop 9a, pop 9b; F_ST_ = −0.03 p = 1.0).

The population divergence within localities was also estimated by AMOVA (see Table 3). Again the variation due to within population polymorphism was greater than between population polymorphism in individual localities indicating a lack of structure. However AMOVA analysis indicated a greater degree of population structure in the locality Cascabel between a palm and chicken hut (pop 16 pop 18; F_ST_ = 0.04, p = 0.01) and Laguna Hermosa (pop 22, pop 23; F_ST_ = 0.02, p = 0.05 and pop 21, pop 22 F_ST_ = 0.06, p = 0.001). F_ST_ comparisons significant in the absence of bonferroni correction.

##### Comparisons within States

###### Portuguesa State

A total of 243 specimens from Portuguesa State were divided into 13 populations and analysed at 9 or 10 microsatellite loci. These included 130 domestic, 92 silvatic and 21 peridomestic specimens. A hierarchical analysis of all populations within Portuguesa detected a greater within population diversity (89%) than between populations (11%), however the associated F_ST_ value indicated structure does exist within the State (F_ST_ = 0.11, p = 0), (Table 3). Pairwise comparisons (F_ST_) indicate that a number of populations contributed to the detected heterogeneity in this State. A domestic population in Santa Lucia (pop 10) was different from the many of populations in Portuguesa possibly due to genetic drift (F_ST_ range = 0.13 to 0.42). Three microsatellite loci were monomorphic in this population and the mean number of alleles and allele richness was the lowest in the study (1.9 and 1.7). Domestic populations in the locality San Bartolo (pop 9a, 9b) were also different from the majority of other populations in Portuguesa (F_ST_ range = 0.04 to 0.26). Both populations were monomorphic at the two loci. Pairwise population comparisons (F_ST_) indicated that geneflow also occurred between localities for example between a house in the locality Terronal and a palm in Palo Gacho (pop 2 and pop 8; F_ST_ = 0.002 p = 0.50). These results were also supported by *cytb* analysis.

When populations in Portuguesa were further analysed in ecotope groupings variation was greatest within populations (88%) in comparison to among groups (0.9%) or among populations within groups (11%) (F_ST_ = 0.12 p = 0, F_SC_ = 0.11 p = 0, F_CT_ = 0.01 p = 2; AMOVA; 2 groups; house <pop 1,2,4,6,9a,9b,10,13>, palm/ chicken hut <3,5,7,8, 35>).

###### Barinas State

A total of 221 specimens from Barinas State were divided into 16 populations and analysed at 10 microsatellite loci. These specimens included 60 domestic, 54 peridomestic and 107 silvatic specimens. Average allele richness was greater in Barinas State (3.1) than Portuguesa (2.3). Expected heterozygosity was higher and ranged from 0.5 to 0.6 (Table 2). A hierarchical analysis of all populations within Barinas detected a greater within population diversity (97%) than between populations diversity (3.3%), however structure does exist within the State (F_ST_ = 0.03, p = 0), (Table 3). When geneflow was examined by pairwise F_ST_ comparisons detected structure was primarily related to a peridomestic population in the locality 19 Abril (pop 26; F_ST_ range = 0.06 to 0.18; see Table S4) in agreement with *cytb* analysis.

When populations in Barinas were further analysed in ecotope groupings variation was greatest within populations (97%) in comparison to among groups (−0.03%) or among populations within groups (3.3%) (F_ST_ = 0.03 p = 0.5, F_SC_ = 0.03 p = 0, F_CT_ = −0.003 p = 5; AMOVA house <17,19,21,24a,24b> palm <18,20,23,24c,24d,25,28> chicken hut <16,22,26,27>).

##### Comparisons between States

###### Lara State

A single domestic population was analysed in this State (pop 31). Mean allele number and richness were low (2.2, 2.0). This population was different by pairwise comparisons from the majority of populations analysed (F_ST_ range = 0.07 to 0.33).

###### Cojedes State

The single domestic and silvatic population from the locality Las Quebralitas also differed from the majority other populations in the study (F_ST_ range = 0.08 to 0.35).

###### Trujillo State

The domestic population analysed from Trujillo (pop 33) wa distinct from the majority of populations (F_ST_ range = 0.09 to 0.42). Four private alleles were detected in this population, all in the single female adult identified as *R. robustus* by *cytb* analysis.

A hierarchical analysis of all 33 populations analysed by microsatellites revealed a greater level of polymorphism within (89%) than between populations (11%), however population structure was detected (F_ST_ = 0.11, p = 0, Table 3). When specimens were grouped by their State of collection (1 group; Portuguesa, Barinas, Cojedes, Trujillo, Lara) detected variation was greater within individual States than between States (7.3%), however State groups were distinct (F_ST_ = 0.07 p = 0) (Table 3).

Additional hierarchal analysis between States was carried out (4 groups; Portuguesa <pop 1–10,13,35>, Barinas <pop 16–28> Cojedes <pop 29,30>, Other <pop 31, 33>). Again variation was greatest within populations (87%) in comparison to among populations within groups (8.3%) or among groups (4.3%) (F_ST_ = 0.13 p = 0, F_SC_ = 0.09 p = 0, F_CT_ = 0.04 p = 0).

#### Isolation by distance (IBD)

Tests for IBD (F_ST_ /(1−F_ST_) against log transformed (ln) distances were conducted at various hierarchical levels (1) between populations (2) between localities (3) within Portuguesa and within Barinas State and (4) between States. Patterns were weakly correlated but significant at population level (33 groups; R2 = 0.06 p-value = 0.0001), locality level (17 groups; R2 = 0.06 p-value = 0.0001) and non-significant at State level (5 groups; R2 = 0.01 p value = 0.64). Patterns were weakly correlated but significant within Portuguesa State (13 groups; R2 = 0.07 p-value = 0.01), within Barinas (16 groups; R2 = 0.02 p-value = 0.01). R2 values range from 0 to 1, with values close to 1 indicating a greater correlation between the compared variables.

## Discussion

National surveys of Chagas disease endemic areas in Venezuela in the 1970s suggested that there were widespread silvatic foci of *R. prolixus,* particularly in palm trees [5]–[8],[36],[37]. It was suggested that such abundant silvatic populations could maintain Chagas transmission by reinvading domestic habitats after vector control campaigns. However, following the identification of the essentially silvatic *R. robustus* in palms in Venezuela, questions were raised as to the epidemiological importance of silvatic *Rhodnius* populations and additionally the taxonomic status of *R. robustus*.

We aimed to resolve the controversy regarding the identity of silvatic populations of *Rhodnius* and the interaction between silvatic and domestic populations, through mitochondrial and microsatellite analyses. Thus our interest and priority here is not in a global analysis of congruence between mitochondrial and microsatellite phylogenetic trees but in applying both methods, with differing resolution to search for continuity between *Rhodnius* populations, particularly between geographically adjacent silvatic and domestic populations. Both methods gave valuable and complementary insight, with different degrees of resolution. A similar picture of shared *cytb* haplotypes and microsatellite homogeneity indicated that silvatic and domestic populations are not isolated, and that gene flow does indeed occur.

### Species identity and genetic relatedness

Mitochondrial DNA has been used previously in triatomine studies, including the tribe Rhodniini [16]–[18]. Here eighteen haplotypes were detected among the 551 Venezuelan specimens analysed and these were confirmed as both *R. prolixus* and *R. robustus* species.

Our data detected silvatic *R. prolixus* in palms in all States, except for Trujillo and Lara. We can therefore unequivocally reaffirm that *R. prolixus* is present in silvatic habitats in Venezuela. Silvatic *R. robustus* does also exist and was the only species detected in this study in palms in Trujillo State (pop 34). In this region the post-spray reinvasion of houses is therefore unlikely, and vector control may be more straightforward. Nevertheless, adult silvatic *R. robustus* have been implicated in the sporadic transmission of *T. cruzi* in western Venezuela [9] and the use of insecticide treated curtains may contribute to reducing sporadic cases of Chagas disease in this State [35].

From sequence analysis it is clear that common haplotypes occur across all ecotopes, with palm and house populations sharing five *R. prolixus* haplotypes. Three of these shared haplotypes were found in domestic nymphs, in addition to domestic adults, thus indicating these silvatic *R. prolixus* are capable of invading and importantly colonising houses.

The incongruence detected between nuclear (D2) and mitochondrial (mt*cytb*) analysis of haplotype 3 confirmed the introgression suspected after the discovery of domestic nymphs of “*R. robustus”*. Introgression has been recorded previously in triatomine species [38] and other haematophagus insects [39],[40]. In accord with colonisation behaviour, these “Amazonian *R. robustus”* are *R. prolixus* with introgressed *R. robustus* mitochondrial DNA. Additional support for introgression is the absence of unique microsatellite alleles in these haplotype 3 specimens, in contrast to our single domestic Venezuelan *R. robustus* adult (haplotype 16), which revealed four unique alleles.

### Genetic variation and population structure

#### Mitochondrial DNA and population structure

In addition to shared haplotypes, population homogeneity was also evident by pairwise comparisons between house, palm and peridomestic sites (pairwise F_ST_ and AMOVA). This includes examples of geneflow between five adjacent ecotopes, also within localities in both Barinas and Portuguesa State. These results indicate that bugs are moving between houses and between palms, in addition to between palms and houses. Importantly, this is supported by recent data analysis from Sanchez-Martin *et al.,* (2006) where infested palms (>10 palms) within 100m of a house were identified as risk factors for house and peridomestic infestation, in addition to palm roofs less than one year old [41]. Additionally a recent morphometric study in Barinas State comparing silvatic populations of *R. prolixus* with pre- and postspray peridomestic and domestic populations was unable to differentiate the silvatic specimens as a separate subpopulation [42]. These results also suggest that silvatic populations of *R. prolixus* are capable of invasion and colonisation and a threat to effective vector control.

When all 34 populations were compared structure was detected (AMOVA, F_ST_ = 0.44). Both pairwise F_ST_ and AMOVA analysis suggest that population heterogeneity was more pronounced within Portuguesa State (39% between population variation) than Barinas (15% between population variation) (Table 3). Interestingly hierarchical analysis indicated that a populations' ecotope is not a factor in determining population differentiation within both Portuguesa and Barinas States (F_CT_ = −0.1, F_CT_ = 0.001). Additionally detected within and among populations variance did not differ greatly between the comparisons all 33 population or populations in an ecotope group hierarchy. This suggests gene flow occurs between populations from different ecotopes. AMOVA analysis of *cytb* data also suggested that detected heterogeneity is not related to the State of origin of a population. Again detected within and among populations variance did not differ greatly between the comparison of all populations or populations in a State group hierarchy.

#### Microsatellite analysis and population structure

For higher resolution of relationships between silvatic and domestic populations of *R. prolixus* in Venezuela a panel of microsatellite markers was developed [29]. Microsatellites are suitable for population genetics and have proven to be highly polymorphic in species with low isoenzyme polymorphism [43],[44], as noted for *R. prolixus* [15]. Nine or ten loci were used; additional loci would be advantageous. Polymorphism was low to moderate for the majority of loci; and excess homozygosity at loci such as LIST14-017 may indicate null alleles, which might hide some diversity at that locus or a Walhund effect with restricted genetic exchange between grouped subpopulations.

As for *cytb* analysis population homogeneity was evident with non-significant pairwise comparisons detected between house, palm and peridomestic sites including adjacent ecotopes. However, some additional genetic diversity was revealed by microsatellites analysis. In the locality Las Queseras populations from an adjacent house and palm (pop 29, pop 30) were significantly different by microsatellites but not by *cytb* analysis. Additionally in the locality Terronal, a house (pop 2) and an adjacent palm (pop 3), and adjacent palms (pop 3, pop 5) were different by microsatellite analysis but indistinguishable by *cytb* analysis. AMOVA analysis also detected further structure between an adjacent palm and house (pop 6, pop 7) not evident in pairwise F_ST_ comparisons which are corrected for multiple comparisons.

Population homogeneity was detected between populations within localities in Portuguesa and Barinas. Both pairwise F_ST_ analysis and AMOVA analysis detected population homogeneity between palm and houses e.g. in the locality Cascabel (pop 17 and pop 18) and in Laguna Hermosa (pop 21, 23), between houses e.g. pop 9a, 9b in the locality San Bartolo, and between palms in the locality G. Paguey (pop 24c, 24d). These results can be explained by the movement of bugs not only between palms and houses but also between houses and between palms.

Comparisons over wider geographic areas revealed population structure (AMOVA, F_ST_). Population structure was detected between all 33 populations (AMOVA, F_ST_ = 0.11). Distinct populations (pairwise F_ST_) exhibited monomorphic loci and low allele richness, suggesting isolation and possible genetic drift. Hierarchical analysis also indicated that population heterogeneity was more pronounced within Portuguesa State (11% between population variation) than Barinas State (3% between population variation) (Table 3). In Portuguesa State populations were collected in mountainous terrain, possibly allowing for greater population isolation, this is in contrast to Barinas, where all localities were situated in flat lands, the Llanos, which could allow for easier mixing of populations. Heterogeneity within Barinas State was primarily related to a single peridomestic population (pop 26; pairwise F_ST_). This population was situated at the extreme distribution of sampled sites in Barinas and in an area where *T. maculata* infestations were more common, factors which may have contributed to detected genetic isolation. The separation of the domestic *R. prolixus* population from Trujillo State (pop 34) from all other populations indicates that the Andes mountain range and the predominance of silvatic *R. robustus* may also act as barriers to gene flow.

Hierarchical analysis of microsatellite data also indicated that population ecotope is not a factor in determining population differentiation within both Portuguesa and Barinas State (F_CT_ = 0.01, F_CT_ = −0.003), thus suggesting geneflow occurs between populations from different ecotopes. Interestingly microsatellite analysis detected greater heterogeneity between populations from different State (F_CT_ = 0.04) as compared to *cytb* analysis.

We investigate the relationship between genetic isolation and increasing geographic distances (IBD). However, while the relationship was significant between populations, between localities and within States, distance was not a critical factor influencing genetic differentiation as the detected correlations were very weak.

As expected a higher degree of population heterogeneity was detected with microsatellites than with the analysis of *cytb* sequences. Microsatellites are fast-evolving, neutral, noncoding loci, whereas the *cytb* is a protein-coding gene with important metabolic functions and thus may be subject to selective constraints [45]. Importantly, populations analysed from different ecotopes and localities, including Terronal, San Bartolo were homogeneous by both methods and distinct populations were also detected by both methods (Trujillo, Santa Lucia and 19 Abril). Occasionally microsatellites uncovered diversity not apparent by *cytb* typing e.g. pop 29, pop 30. Both or pairwise F_ST_ and AMOVA data for both methods are consistent with movement between silvatic and domestic habitats with ecotope not determining population structure and with greater population heterogeneity in Portuguesa than Barinas State.

Our results contrast a recent microsatellite study of 19 populations of *T. infestans* from domestic and peridomestic ecotopes in Argentina. The analysis indicated a strong population structure, with limited gene flow and genetic drift leading to genetic differentiation and suggested an important role for recrudescence in post control infestations rather than reinvasion from untreated areas [46].

### Conclusions

Movement of bugs between silvatic, peridomestic and domestic ecotopes probably occurs both actively and passively. Risk factor analysis detected an association between new thatched palm roofs and infestation [41]. Female *R. prolixus* glue their eggs to palm fronds suggesting passive transport of bugs into houses on these fronds [6]. Restriction or elimination of palm roofs on dwellings must therefore be a key element of control strategies, although it is important that an appropriate substitute roofing material is readily available to the inhabitants. Active transport can also occur, flying adult triatomine bugs may enter a house attracted to light [9]. *Rhodnius prolixus* in Venezuela is known to be light attracted [47].

From our data it is clear that silvatic populations of *R. prolixus* in Venezuela represent a definite threat to successful control of Chagas disease, as suspected but controversially debated since populations of *R. prolixus* were reported in palm trees [5]. Results indicate that the current control programme in Venezuela is unlikely to achieve the level of success seen in the Southern cone, where *T. infestans* has been eliminated over large areas [1]. The control programme will have to deal with this continual threat, for example by more frequent spraying of houses, combined with community vigilance for reinfestations as an integral part of the control programme. The additional use of alternative control methods such as insecticide treated curtains [35] or bednets [48] would be beneficial. Increased housing improvements, although expensive, seem vital for long term control, by creating a domestic environment unsuitable for colonisation by silvatic bugs.

This study has made a fundamental contribution to the understanding of *Rhodnius* populations in the context of disease epidemiology and vector control in Venezuela. An important follow-up to this project would be to define population interaction more extensively, particularly in regions of Colombia, where silvatic and domestic *Rhodnius* populations also occur and reinvasion may be maintaining large domestic colonies of *R. prolixus* [49]. This would allow prioritisation of control interventions and tailoring of control strategies to regional circumstances. Additionally, modified control strategies to counteract the threat of reinvasion could be assessed, such as widespread provision of ideal low cost roofing, the treatment or removal of palms close to houses, and, improved spraying and surveillance, all with the aim of reducing the burden of Chagas disease in rural areas.

## Supporting Information

Table S1 The pairwise comparison of 34 populations from six Venezuelan States by cytb analysis; F_ST_ values below diagonal (p-values above) (Arlequin v3.1). Values in bold remain significant following sequential Bonferroni correction (k = 561, p1 = 0.05/561, p≤0.0001). See Table 1 for population details.
(0.05 MB PDF)

Table S2 Summary of population microsatellite data. (A) Summary of population microsatellite data per locus (LIST14-056, LIST14-017, LIST14-042, LIST14-010, LIST14-064). N = number of specimens amplified, N_A_ = number of alleles, H_O_, H_E_ = Observed and Expected heterozygosity, P = exact probability for expected Hardy Weinberg equilibrium conditions for each locus/population combination (Arlequin v2.1), M = monomorphic. F_IS_ = Weir & Cockerham (1984) (GENEPOP V3.4). Values in bold departures from HWE significant after Bonferroni correction (populations analysed at 9 loci k = 9, p1 = 0.05/9, at 10 loci k = 10, p1 = 0.05/10). See Table 2 for population details. (B) Summary of population microsatellite data per locus (LIST14-013, LIST14-021, LIST14-025, LIST14-037, LIST14-079). N = number of specimens amplified, N_A_ = number of alleles, H_O_, H_E_ = Observed and Expected heterozygosity, P = exact probability for expected Hardy Weinberg equilibrium conditions for each locus/population combination (Arlequin v2.1). F_IS_ = Weir & Cockerham (1984) (GENEPOP V3.4). Values in bold departures from HWE significant after Bonferroni correction, populations analysed (9 loci k = 9, p1 = 0.05/9, at 10 loci k = 10, p1 = 0.05/10). ^∧^LIST14-079 amplified in subset of populations. M = monomorphic, NA = not amplified. See Table 2 for population details.
(0.02 MB PDF)

Table S3 The pairwise comparison of 33 populations from six Venezuelan States at 9 microsatellite loci, FST values below diagonal (p-values above) (Arlequin v2.1). Values in bold significant after sequential Bonferroni correction k = 528, p1 = 0.05/528, p≤0.0001. See Table 2 for population details.
(0.05 MB PDF)

Table S4 The pairwise comparison of a subset of 20 populations at 10 microsatellite loci FST values below diagonal (p-values above) (Arlequin v2.1). Values in bold significant after sequential Bonferroni correction k = 190, p1 = 0.05/190, p≤0.0003.
(0.04 MB PDF)
