# Supplementary material for: Molecular Genetics Reveal That Silvatic Rhodnius prolixus Do Colonise Rural Houses
Source: PLoS Negl Trop Dis. 2008 Apr 2;2(4):e210. doi: 10.1371/journal.pntd.0000210 (PMC2270345; doi:10.1371/journal.pntd.0000210)
Supplement: Table S1 — The pairwise comparison of 34 populations from six Venezuelan States by cytb analysis; FST values below diagonal (p-values above) (Arlequin v3.1). Values in bold remain significant following sequential Bonferroni correction (k = 561, p1 = 0.05/561, p≤0.0001). See Table 1 for population details. (0.05 MB PDF) [file pntd.0000210.s001.pdf]

Table S1 The pairwise comparison of 34 populations from six Venezuelan States by cytb analysis; FST values below diagonal (p-values above) (Arlequin v3.1).

	pop 1	pop 2	pop 3	pop 4	pop 5	pop 6	pop 7	pop 8	pop 9	pop 10	pop 11	pop 12	pop 13	pop 14	pop 15	pop 16	pop 17
**pop 1**		0.0216	**0.0000**	0.7144	0.0002	**0.0000**	0.0005	0.2461	**0.0000**	**0.0000**	0.0002	0.0003	0.0703	0.0023	0.0005	0.0010	0.0023
**pop 2**	0.1828		0.1428	0.1837	0.2587	0.0031	0.0840	0.9999	0.0006	0.0073	0.0321	0.0796	0.0002	0.1505	0.0828	0.0693	0.0756
**pop 3**	0.4268	0.0470		0.0061	0.7674	0.0688	0.2597	0.1846	0.0188	0.0113	0.2092	0.2666	**0.0000**	0.5057	0.3551	0.1448	0.3451
**pop 4**	-0.0343	0.0593	0.3020		0.0158	**0.0000**	0.0063	0.6830	**0.0000**	0.0003	0.0016	0.0061	0.0489	0.0176	0.0122	0.0077	0.0175
**pop 5**	0.3665	0.0130	-0.0238	0.2361		0.0352	0.1755	0.2205	0.0139	0.0311	0.1300	0.1828	**0.0000**	0.3314	0.1920	0.1954	0.3170
**pop 6**	0.6617	0.2766	0.0968	0.6025	0.1324		0.5542	0.0082	0.1831	**0.0000**	0.0601	0.5509	**0.0000**	0.9999	0.0013	0.0313	0.6640
**pop 7**	0.5813	0.2035	0.0522	0.4903	0.0820	-0.0046		0.0837	0.9999	0.0007	0.2201	0.9999	**0.0000**	0.9999	0.0477	0.0347	0.1827
**pop 8**	0.0854	-0.0630	0.0959	-0.0312	0.0414	0.4704	0.3333		0.0024	0.0037	0.0265	0.0874	0.0112	0.1019	0.1000	0.0865	0.0936
**pop 9**	0.7012	0.3171	0.1212	0.6551	0.1596	0.0666	0.0000	0.5383		**0.0000**	0.0039	0.9999	**0.0000**	0.9999	0.0003	0.0018	0.0445
**pop 10**	0.6219	0.2518	0.1210	0.5506	0.1406	0.6297	0.6276	0.4126	0.7537		**0.0000**	0.0007	**0.0000**	0.0043	**0.0000**	**0.0001**	0.0008
**pop 11**	0.5848	0.2102	0.0626	0.4961	0.0909	0.0976	0.0574	0.3389	0.1927	0.4351		0.2144	**0.0000**	0.4440	0.1962	0.1347	0.9035
**pop 12**	0.5813	0.2035	0.0522	0.4903	0.0820	-0.0046	0.0000	0.3333	0.0000	0.6276	0.0574		0.0002	0.9999	0.0516	0.0335	0.1793
**pop 13**	0.1971	0.5511	0.7536	0.3349	0.7053	0.9958	1.0000	0.5556	1.0000	0.9900	0.9798	1.0000		**0.0000**	**0.0000**	**0.0000**	**0.0000**
**pop 14**	0.5497	0.1711	0.0251	0.4437	0.0541	-0.0339	0.0000	0.2746	0.0000	0.5922	0.0163	0.0000	1.0000		0.4075	0.0886	0.9999
**pop 15**	0.4815	0.1053	-0.0034	0.3648	0.0137	0.1289	0.0671	0.1770	0.1999	0.2165	0.0129	0.0671	0.8846	0.0269		0.6174	0.4948
**pop 16**	0.5565	0.1826	0.0458	0.4569	0.0729	0.2457	0.3187	0.2931	0.5330	0.5444	0.0760	0.3187	0.9872	0.2566	-0.0183		0.2913
**pop 17**	0.5534	0.1754	0.0298	0.4504	0.0589	-0.0197	0.0295	0.2829	0.1888	0.5149	-0.0631	0.0295	0.9924	-0.0182	-0.0107	0.0382	
**pop 18**	0.6031	0.2256	0.0750	0.5228	0.1046	0.1659	0.2007	0.3715	0.3590	0.4948	0.0534	0.2007	0.9810	0.1568	0.0147	-0.0879	0.0091
**pop 19**	0.5226	0.1374	-0.0081	0.4026	0.0210	-0.0706	0.0000	0.2208	0.0000	0.5619	-0.0280	0.0000	1.0000	0.0000	-0.0165	0.1995	-0.0687
**pop 20**	0.5908	0.2122	0.0585	0.5045	0.0892	-0.0186	0.0410	0.3496	0.1689	0.5252	0.0408	0.0410	0.9911	0.0012	0.0494	0.0599	-0.0567
**pop 21**	0.5878	0.2097	0.0571	0.4998	0.0869	0.0251	-0.0092	0.3447	0.0991	0.5848	-0.0055	-0.0092	0.9967	-0.0462	0.0539	0.2549	-0.0574
**pop 22**	0.5435	0.1688	0.0310	0.4355	0.0572	0.2118	0.1759	0.2655	0.4013	0.5085	-0.0947	0.1759	0.9895	0.1111	-0.0179	0.1634	-0.0355
**pop 23**	0.5574	0.1824	0.0408	0.4573	0.0687	0.1060	0.1155	0.2920	0.2993	0.4698	-0.0485	0.1155	0.9837	0.0632	-0.0265	-0.0286	-0.0838
**pop 24**	0.5534	0.1754	0.0298	0.4504	0.0589	-0.0197	0.0295	0.2829	0.1888	0.5149	-0.0631	0.0295	0.9924	-0.0182	-0.0107	0.0382	-0.1429
**pop 25**	0.5777	0.2038	0.0572	0.4850	0.0855	0.1148	0.0741	0.3268	0.2324	0.5139	0.0698	0.0741	0.9905	0.0278	0.0702	0.2237	0.0389
**pop 26**	0.5995	0.2626	0.1727	0.5175	0.1815	0.8918	1.0000	0.3862	1.0000	0.8394	0.6365	1.0000	1.0000	1.0000	0.2830	0.7619	0.8215
**pop 27**	0.5314	0.1500	0.0057	0.4174	0.0350	-0.0845	0.0909	0.2409	0.3101	0.5356	-0.0183	0.0909	0.9959	0.0278	-0.0212	0.0085	-0.1180
**pop 28**	0.5637	0.1859	0.0390	0.4658	0.0686	-0.0042	0.1411	0.3024	0.3327	0.5470	0.0322	0.1411	0.9936	0.0870	0.0159	-0.0053	-0.0651
**pop 29**	0.6667	0.2826	0.1024	0.6095	0.1375	0.0476	0.0000	0.4811	0.0000	0.7191	0.1524	0.0000	1.0000	0.0000	0.1603	0.4737	0.1415
**pop 30**	0.5347	0.1350	-0.0070	0.4313	0.0168	0.0331	-0.0103	0.2356	0.0553	0.1304	0.0113	-0.0103	0.8790	-0.0388	-0.0097	0.0137	-0.0292
**pop 31**	0.6774	0.2930	0.1081	0.6237	0.1442	0.0534	0.0000	0.4990	0.0000	0.7299	0.1645	0.0000	1.0000	0.0000	0.1722	0.4922	0.1558
**pop 32**	0.6405	0.2664	0.1166	0.5746	0.1448	0.3662	0.4004	0.4374	0.5426	0.5791	0.2693	0.4004	0.9814	0.3629	0.0933	-0.0094	0.2310
**pop 33**	0.6415	0.2937	0.1836	0.5769	0.1963	0.5655	0.5050	0.4499	0.6390	0.6105	0.2818	0.5050	0.9710	0.4697	0.1225	0.4203	0.3739
**pop 34**	0.6319	0.4989	0.5973	0.5756	0.5600	0.9750	0.9751	0.5381	0.9893	0.9587	0.9220	0.9751	0.9906	0.9684	0.7267	0.9332	0.9477

	pop 18	pop 19	pop 20	pop 21	pop 22	pop 23	pop 24	pop 25	pop 26	pop 27	pop 28	pop 29	pop 30	pop 31	pop 32	pop 33	pop 34
**pop 1**	**0.0000**	0.0100	**0.0001**	0.0005	0.0025	0.0005	0.0026	**0.0000**	**0.0000**	0.0055	0.0005	**0.0000**	**0.0000**	**0.0000**	**0.0000**	**0.0000**	**0.0000**
**pop 2**	0.0174	0.2950	0.0337	0.0398	0.1142	0.0741	0.0847	0.0441	0.0060	0.1540	0.0749	0.0020	0.0397	0.0017	0.0050	0.0002	0.0023
**pop 3**	0.0785	0.5184	0.3103	0.2686	0.2452	0.2046	0.3445	0.0971	0.0151	0.5708	0.3352	0.0506	0.3749	0.0526	0.0194	**0.0010**	**0.0001**
**pop 4**	0.0008	0.0475	0.0019	0.0031	0.0176	0.0080	0.0165	0.0029	0.0029	0.0410	0.0060	0.0002	0.0018	0.0002	**0.0001**	**0.0000**	0.0009
**pop 5**	0.0477	0.5656	0.1739	0.1685	0.0880	0.2701	0.3111	0.0337	0.0313	0.3742	0.3136	0.0357	0.2833	0.0347	0.0085	0.0081	0.0002
**pop 6**	0.0141	0.9999	0.4347	0.2502	0.0596	0.0984	0.6605	0.0362	**0.0000**	0.9999	0.5621	0.4805	0.4894	0.2222	**0.0001**	**0.0000**	**0.0000**
**pop 7**	0.0340	0.9999	0.4795	0.9999	0.1639	0.0867	0.1839	0.4709	**0.0000**	0.3833	0.2066	0.9999	0.5714	0.9999	**0.0010**	**0.0000**	**0.0003**
**pop 8**	0.0157	0.2275	0.0304	0.0350	0.1019	0.0864	0.0928	0.0431	**0.0000**	0.2319	0.0921	0.0061	0.0453	0.0045	0.0033	0.0012	0.0011
**pop 9**	**0.0000**	0.9999	0.0773	0.2725	0.0318	0.0101	0.0470	0.0142	**0.0000**	0.1696	0.0496	0.9999	0.2026	0.9999	**0.0000**	**0.0000**	**0.0000**
**pop 10**	**0.0000**	0.0081	**0.0000**	0.0003	0.0002	**0.0000**	0.0005	**0.0001**	**0.0000**	0.0024	**0.0001**	**0.0000**	0.0004	**0.0000**	**0.0000**	**0.0000**	**0.0000**
**pop 11**	0.1756	0.6798	0.3901	0.5859	0.9999	0.8172	0.9028	0.0669	**0.0000**	0.7108	0.2861	0.0104	0.1240	0.0073	0.0005	**0.0000**	0.0002
**pop 12**	0.0363	0.9999	0.4823	0.9999	0.1508	0.0855	0.1857	0.4782	**0.0001**	0.3718	0.2101	0.9999	0.5841	0.9999	0.0013	**0.0000**	**0.0001**
**pop 13**	**0.0000**	**0.0005**	**0.0000**	**0.0000**	**0.0000**	**0.0000**	**0.0000**	**0.0000**	**0.0000**	**0.0001**	**0.0000**	**0.0000**	**0.0000**	**0.0000**	**0.0000**	**0.0000**	**0.0001**
**pop 14**	0.1013	0.9999	0.5077	0.9999	0.4652	0.2133	0.9999	0.4850	**0.0001**	0.4608	0.4805	0.9999	0.9999	0.9999	0.0058	**0.0000**	0.0005
**pop 15**	0.2358	0.4151	0.0440	0.0524	0.4669	0.6475	0.5048	0.0244	**0.0000**	0.4224	0.3415	0.0010	0.5445	0.0005	0.0050	**0.0001**	**0.0001**
**pop 16**	0.9999	0.2192	0.3200	0.0265	0.0604	0.6930	0.2934	0.0147	**0.0001**	0.5730	0.6140	0.0038	0.0705	0.0023	0.3441	**0.0000**	**0.0000**
**pop 17**	0.3709	0.9999	0.8229	0.7058	0.4894	0.9999	0.9999	0.3234	**0.0001**	0.9999	0.7892	0.0650	0.7753	0.0559	0.0246	**0.0001**	**0.0002**
**pop 18**		0.2987	0.1566	0.0410	0.1394	0.6371	0.3600	0.0143	**0.0000**	0.5331	0.4630	**0.0001**	0.1467	**0.0000**	0.1312	**0.0000**	**0.0000**
**pop 19**	0.1138		0.7769	0.9999	0.4727	0.4136	0.9999	0.6774	**0.0001**	0.9999	0.5046	0.9999	0.9999	0.9999	0.0417	0.0032	0.0022
**pop 20**	0.0413	-0.0426		0.3590	0.1324	0.3995	0.8199	0.1247	**0.0000**	0.9999	0.9999	0.1141	0.3382	0.1025	0.0106	**0.0000**	**0.0001**
**pop 21**	0.1661	-0.0891	0.0404		0.4288	0.3155	0.6948	0.1452	**0.0000**	0.6007	0.1830	0.3378	0.5522	0.3117	0.0006	**0.0000**	**0.0000**
**pop 22**	0.1042	0.0492	0.1116	0.0284		0.5939	0.4984	0.1029	**0.0000**	0.3314	0.0854	0.0515	0.4398	0.0476	0.0017	0.0026	0.0003
**pop 23**	-0.0294	0.0110	0.0063	0.0419	-0.0320		0.9999	0.0431	**0.0000**	0.7756	0.7891	0.0209	0.0844	0.0148	0.0246	**0.0000**	0.0002
**pop 24**	0.0091	-0.0687	-0.0567	-0.0574	-0.0355	-0.0838		0.3321	**0.0000**	0.9999	0.7949	0.0659	0.7766	0.0538	0.0251	**0.0001**	0.0003
**pop 25**	0.1812	-0.0204	0.0698	0.0627	0.1046	0.0951	0.0389		0.0009	0.3794	0.1699	0.0250	0.0918	0.0183	0.0003	**0.0000**	0.0003
**pop 26**	0.6760	1.0000	0.7965	0.9126	0.7775	0.6928	0.8215	0.6914		**0.0000**	**0.0000**	**0.0000**	0.0214	**0.0000**	**0.0000**	**0.0000**	**0.0001**
**pop 27**	-0.0143	-0.0345	-0.1220	0.0257	0.0690	-0.0546	-0.1180	0.0291	0.8947		0.9999	0.2190	0.9999	0.2025	0.0810	0.0008	0.0014
**pop 28**	-0.0134	0.0337	-0.0801	0.0987	0.1297	-0.0227	-0.0651	0.0932	0.8491	-0.1501		0.0744	0.3997	0.0666	0.0923	**0.0000**	0.0002
**pop 29**	0.3117	0.0000	0.1309	0.0678	0.3371	0.2450	0.1415	0.1852	1.0000	0.2466	0.2764		0.4864	0.9999	**0.0000**	**0.0000**	**0.0000**
**pop 30**	0.0394	-0.0749	0.0010	-0.0047	-0.0195	-0.0068	-0.0292	0.0014	0.2252	-0.0548	-0.0161	0.0383		0.4942	**0.0000**	**0.0000**	**0.0000**
**pop 31**	0.3260	0.0000	0.1424	0.0773	0.3569	0.2615	0.1558	0.1995	1.0000	0.2661	0.2936	0.0000	0.0435		**0.0000**	**0.0000**	**0.0000**
**pop 32**	0.0403	0.3288	0.2127	0.3870	0.3550	0.1640	0.2310	0.3579	0.7181	0.1727	0.1494	0.5005	0.1146	0.5134		**0.0000**	**0.0000**
**pop 33**	0.4098	0.4387	0.4774	0.4442	0.2542	0.3028	0.3739	0.4696	0.6867	0.4407	0.4702	0.6001	0.2257	0.6121	0.5469		**0.0000**
**pop 34**	0.9279	0.9616	0.9513	0.9672	0.9374	0.9248	0.9477	0.9478	0.9769	0.9514	0.9532	0.9864	0.7393	0.9874	0.9331	0.9103	

Values in bold remain significant following sequential Bonferroni correction (k=561, p1=0.05/561 ≤0.0001). see Table 1 for population details

Table S1 The standard deviation of P values (Arlequin v3.1).

	pop 1	pop 2	pop 3	pop 4	pop 5	pop 6	pop 7	pop 8	pop 9	pop 10	pop 11	pop 12	pop 13	pop 14	pop 15	pop 16	pop 17	pop 18	pop 19	pop 20	pop 21	pop 22	pop 23	pop 24	pop 25	pop 26	pop 27	pop 28	pop 29	pop 30	pop 31	pop 32	pop 33	pop 34
**pop 1**		±0.0014	**±0.0000**	±0.0047	±0.0001	**±0.0000**	±0.0002	±0.0040	**±0.0000**	**±0.0000**	±0.0001	±0.0002	±0.0025	±0.0005	±0.0003	±0.0003	±0.0005	**±0.0000**	±0.0010	**±0.0001**	±0.0002	±0.0005	±0.0003	±0.0005	**±0.0000**	**±0.0000**	±0.0008	±0.0002	**±0.0000**	**±0.0000**	**±0.0000**	**±0.0000**	**±0.0000**	**±0.0000**
**pop 2**			±0.0035	±0.0036	±0.0049	±0.0005	±0.0025	±0.0000	±0.0002	±0.0009	±0.0017	±0.0032	±0.0001	±0.0036	±0.0025	±0.0028	±0.0025	±0.0014	±0.0043	±0.0020	±0.0018	±0.0029	±0.0025	±0.0026	±0.0018	±0.0007	±0.0034	±0.0025	±0.0004	±0.0019	±0.0004	±0.0007	±0.0001	±0.0005
**pop 3**				±0.0008	±0.0040	±0.0024	±0.0041	±0.0037	±0.0013	±0.0010	±0.0044	±0.0043	**±0.0000**	±0.0053	±0.0049	±0.0035	±0.0057	±0.0024	±0.0052	±0.0046	±0.0043	±0.0044	±0.0038	±0.0044	±0.0027	±0.0011	±0.0050	±0.0047	±0.0021	±0.0049	±0.0025	±0.0014	**±0.0004**	**±0.0001**
**pop 4**					±0.0012	**±0.0000**	±0.0008	±0.0047	**±0.0000**	±0.0002	±0.0004	±0.0008	±0.0018	±0.0012	±0.0011	±0.0008	±0.0013	±0.0003	±0.0019	±0.0004	±0.0006	±0.0015	±0.0010	±0.0013	±0.0006	±0.0006	±0.0020	±0.0007	±0.0001	±0.0004	±0.0001	**±0.0001**	**±0.0000**	±0.0003
**pop 5**						±0.0018	±0.0039	±0.0039	±0.0012	±0.0016	±0.0031	±0.0039	**±0.0000**	±0.0048	±0.0036	±0.0040	±0.0046	±0.0022	±0.0048	±0.0037	±0.0036	±0.0023	±0.0045	±0.0047	±0.0020	±0.0019	±0.0041	±0.0042	±0.0019	±0.0043	±0.0017	±0.0010	±0.0009	±0.0001
**pop 6**							±0.0056	±0.0009	±0.0037	**±0.0000**	±0.0023	±0.0052	**±0.0000**	±0.0000	±0.0003	±0.0019	±0.0045	±0.0013	±0.0000	±0.0049	±0.0046	±0.0025	±0.0031	±0.0046	±0.0019	**±0.0000**	±0.0000	±0.0049	±0.0054	±0.0051	±0.0043	**±0.0001**	**±0.0000**	**±0.0000**
**pop 7**								±0.0024	±0.0000	±0.0003	±0.0041	±0.0000	**±0.0000**	±0.0000	±0.0021	±0.0019	±0.0038	±0.0019	±0.0000	±0.0049	±0.0000	±0.0032	±0.0028	±0.0035	±0.0048	**±0.0000**	±0.0053	±0.0043	±0.0000	±0.0051	±0.0000	**±0.0003**	**±0.0000**	**±0.0002**
**pop 8**									±0.0004	±0.0006	±0.0017	±0.0026	±0.0011	±0.0031	±0.0029	±0.0030	±0.0029	±0.0011	±0.0041	±0.0017	±0.0018	±0.0026	±0.0029	±0.0029	±0.0020	**±0.0000**	±0.0049	±0.0030	±0.0008	±0.0020	±0.0007	±0.0006	±0.0004	±0.0004
**pop 9**										**±0.0000**	±0.0006	±0.0000	**±0.0000**	±0.0000	±0.0002	±0.0004	±0.0020	**±0.0000**	±0.0000	±0.0027	±0.0045	±0.0018	±0.0011	±0.0021	±0.0012	**±0.0000**	±0.0040	±0.0020	±0.0000	±0.0036	±0.0000	**± 0.0000**	**±0.0000**	**±0.0000**
**pop 10**											**±0.0000**	±0.0003	**±0.0000**	±0.0007	**±0.0000**	**±0.0001**	±0.0003	**±0.0000**	±0.0009	**±0.0000**	±0.0002	±0.0001	**±0.0000**	±0.0002	**±0.0001**	**±0.0000**	±0.0005	**±0.0001**	**±0.0000**	±0.0002	**±0.0000**	**± 0.0000**	**±0.0000**	**±0.0000**
**pop 11**												±0.0036	**±0.0000**	±0.0048	±0.0043	±0.0032	±0.0029	±0.0037	±0.0048	±0.0050	±0.0046	±0.0000	±0.0036	±0.0029	±0.0022	**±0.0000**	±0.0044	±0.0044	±0.0010	±0.0033	±0.0009	±0.0002	**±0.0000**	±0.0001
**pop 12**													±0.0001	±0.0000	±0.0022	±0.0020	±0.0038	±0.0021	±0.0000	±0.0049	±0.0000	±0.0031	±0.0024	±0.0041	±0.0054	**±0.0001**	±0.0044	±0.0036	±0.0000	±0.0050	±0.0000	±0.0004	**±0.0000**	**±0.0001**
**pop 13**														**±0.0000**	**±0.0000**	**±0.0000**	**±0.0000**	**±0.0000**	**±0.0003**	**±0.0000**	**±0.0000**	**±0.0000**	**±0.0000**	**±0.0000**	**±0.0000**	**±0.0000**	**±0.0001**	**±0.0000**	**±0.0000**	**±0.0000**	**±0.0000**	**± 0.0000**	**±0.0000**	**±0.0001**
**pop 14**															±0.0050	±0.0026	±0.0000	±0.0029	±0.0000	±0.0047	±0.0000	±0.0052	±0.0039	±0.0000	±0.0046	**±0.0001**	±0.0051	±0.0057	±0.0000	±0.0000	±0.0000	±0.0008	**±0.0000**	±0.0003
**pop 15**																±0.0050	±0.0044	±0.0046	±0.0046	±0.0022	±0.0023	±0.0048	±0.0049	±0.0049	±0.0016	**±0.0000**	±0.0045	±0.0049	±0.0003	±0.0052	±0.0002	±0.0007	**±0.0001**	**±0.0001**
**pop 16**																	±0.0048	±0.0000	±0.0044	±0.0051	±0.0017	±0.0025	±0.0041	±0.0043	±0.0012	**±0.0001**	±0.0047	±0.0045	±0.0006	±0.0025	±0.0005	±0.0042	**±0.0000**	**±0.0000**
**pop 17**																		±0.0044	±0.0000	±0.0042	±0.0046	±0.0051	±0.0000	±0.0000	±0.0045	**±0.0001**	±0.0000	±0.0036	±0.0025	±0.0040	±0.0024	±0.0015	**±0.0001**	**±0.0001**
**pop 18**																			±0.0042	±0.0035	±0.0018	±0.0035	±0.0053	±0.0047	±0.0011	**±0.0000**	±0.0051	±0.0051	**±0.0001**	±0.0037	**±0.0000**	±0.0033	**±0.0000**	**±0.0000**
**pop 19**																				±0.0041	±0.0000	±0.0055	±0.0045	±0.0000	±0.0044	**±0.0001**	±0.0000	±0.0043	±0.0000	±0.0000	±0.0000	±0.0019	±0.0005	±0.0004
**pop 20**																					±0.0045	±0.0034	±0.0052	±0.0042	±0.0031	**±0.0000**	±0.0000	±0.0000	±0.0033	±0.0047	±0.0030	±0.0010	**±0.0000**	**±0.0001**
**pop 21**																						±0.0044	±0.0055	±0.0041	±0.0038	**±0.0000**	±0.0046	±0.0038	±0.0047	±0.0046	±0.0039	±0.0002	**±0.0000**	**±0.0000**
**pop 22**																							±0.0044	±0.0047	±0.0032	**±0.0000**	±0.0048	±0.0028	±0.0023	±0.0046	±0.0021	±0.0004	±0.0005	±0.0002
**pop 23**																								±0.0000	±0.0021	**±0.0000**	±0.0036	±0.0037	±0.0015	±0.0028	±0.0012	±0.0014	**±0.0000**	±0.0001
**pop 24**																									±0.0052	**±0.0000**	±0.0000	±0.0038	±0.0025	±0.0041	±0.0022	±0.0015	**±0.0001**	±0.0002
**pop 25**																										±0.0003	±0.0047	±0.0039	±0.0015	±0.0029	±0.0014	±0.0002	**±0.0000**	±0.0002
**pop 26**																											**±0.0000**	**±0.0000**	**±0.0000**	±0.0013	**±0.0000**	**± 0.0000**	**±0.0000**	**±0.0001**
**pop 27**																												±0.0000	±0.0040	±0.0000	±0.0042	±0.0027	±0.0003	±0.0004
**pop 28**																													±0.0027	±0.0041	±0.0024	±0.0031	**±0.0000**	±0.0001
**pop 29**																														±0.0052	±0.0000	**± 0.0000**	**±0.0000**	**±0.0000**
**pop 30**																															±0.0044	**± 0.0000**	**±0.0000**	**±0.0000**
**pop 31**																																**± 0.0000**	**±0.0000**	**±0.0000**
**pop 32**																																	**±0.0000**	**±0.0000**
**pop 33**																																		**±0.0000**
**pop 34**																																		
