# Supplementary material for: Molecular Genetics Reveal That Silvatic Rhodnius prolixus Do Colonise Rural Houses
Source: PLoS Negl Trop Dis. 2008 Apr 2;2(4):e210. doi: 10.1371/journal.pntd.0000210 (PMC2270345; doi:10.1371/journal.pntd.0000210)
Supplement: Table S2 — Summary of population microsatellite data. (A) Summary of population microsatellite data per locus (LIST14-056, LIST14-017, LIST14-042, LIST14-010, LIST14-064). N = number of specimens amplified, NA = number of alleles, HO, HE = Observed and Expected heterozygosity, P = exact probability for expected Hardy Weinberg equilibrium conditions for each locus/population combination (Arlequin v2.1), M = monomorphic. FIS = Weir & Cockerham (1984) (GENEPOP V3.4). Values in bold departures from HWE significant after Bonferroni correction (populations analysed at 9 loci k = 9, p1 = 0.05/9, at 10 loci k = 10, p1 = 0.05/10). See Table 2 for population details. (B) Summary of population microsatellite data per locus (LIST14-013, LIST14-021, LIST14-025, LIST14-037, LIST14-079). N = number of specimens amplified, NA = number of alleles, HO, HE = Observed and Expected heterozygosity, P = exact probability for expected Hardy Weinberg equilibrium conditions for each locus/population combination (Arlequin v2.1). FIS = Weir & Cockerham (1984) (GENEPOP V3.4). Values in bold departures from HWE significant after Bonferroni correction, populations analysed (9 loci k = 9, p1 = 0.05/9, at 10 loci k = 10, p1 = 0.05/10). ∧LIST14-079 amplified in subset of populations. M = monomorphic, NA = not amplified. See Table 2 for population details. (0.02 MB PDF) [file pntd.0000210.s002.pdf]

Table S2. Summary of population microsatellite data

(A) Summary of population microsatellite data per locus (LIST14-056, LIST14-017, LIST14-042, LIST14-010, LIST14-064)

	LIST14-056						LIST14-017					LIST14-042					LIST14-010					LIST14-064				
**Pop**	**N**	**N_A**	**H_O**	**H_E**	**P**	**F_{IS}**	**N_A**	**H_O**	**H_E**	**P**	**F_{IS}**	**N_A**	**H_O**	**H_E**	**P**	**F_{IS}**	**N_A**	**H_O**	**H_E**	**P**	**F_{IS}**	**N_A**	**H_O**	**H_E**	**P**	**F_{IS}**
**1**	26	5	0.39	0.47	0.31	+0.17	4	**0.35**	**0.67**	**0.004**	+0.47	2	0.27	0.34	0.29	+0.21	2	0.08	0.19	0.12	+0.47	2	0.19	0.27	0.37	+0.19
**2**	18	6	0.50	0.61	0.41	+0.12	3	**0.33**	**0.70**	**0.02**	+0.51	2	0.56	0.51	1	-0.08	3	0.22	0.35	0.20	+0.26	2	**0.06**	**0.30**	**0.01**	+0.78
**3**	26	4	0.60	0.54	0.76	-0.11	3	0.16	0.4	**0.001**	+0.62	2	0.08	0.11	1	-0.02	2	0.04	0.08	1	-	2	**0.04**	**0.49**	**<0.0001**	+0.92
**4**	10	3	0.50	0.50	1	-0.18	3	0.40	0.79	0.20	+0.44	2	0.10	0.35	0.16	+0.64	4	0.40	0.36	1	-0.11	2	0.63	0.53	1	-0.21
**5**	39	7	0.68	0.68	0.59	-0.00	4	**0.26**	**0.67**	**<0.0001**	+0.61	2	0.56	0.54	0.54	-0.12	2	0.16	0.20	1	-0.07	3	0.31	0.43	0.31	+0.22
**6**	24	2	0.42	0.38	1	-0.09	3	**0.17**	**0.30**	**0.006**	+0.38	2	0.50	0.50	1	-0.05	5	0.63	0.66	0.75	+0.01	1	M	M	M	M
**7**	12	5	0.67	0.67	1	-0.05	3	0.17	0.53	0.01	+0.64	2	0.75	0.52	0.24	-0.48	4	0.50	0.51	0.12	+0.02	2	0.00	0.26	0.05	+1
**8**	15	6	0.53	0.55	0.50	+0.03	3	0.33	0.60	0.17	+0.39	2	0.27	0.56	0.11	+0.47	2	0.13	0.19	1	-0.04	2	0.20	0.35	0.33	+0.31
**9a**	14	3	0.14	0.27	0.08	+0.47	4	**0.29**	**0.74**	**0.001**	+0.60	2	0.5	0.50	1	-0.01	1	M	M	M	M	1	M	M	M	M
**9b**	14	3	0.21	0.37	0.37	+0.33	4	**0.36**	**0.71**	**0.006**	+0.50	2	0.21	0.52	0.07	+0.54	1	M	M	M	M	1	M	M	M	M
**10**	13	3	**0.77**	**0.62**	**0.004**	-0.35	2	0.08	0.15	1	-	1	M	M	M	M	1	M	M	M	M	1	M	M	M	M
**13**	11	3	0.64	0.54	0.63	-0.35	3	0.82	0.66	0.85	-0.25	2	0.36	0.52	0.54	+0.26	1	M	M	M	M	2	0.18	0.26	1	-0.05
**16**	11	3	0.36	0.39	1	-0.13	3	0.27	0.53	0.14	+0.41	2	0.36	0.54	0.53	+0.26	2	0.20	0.28	1	-0.06	3	0.46	0.46	0.60	+0.00
**17**	10	4	0.80	0.64	0.55	-0.26	3	0.50	0.64	0.09	+0.22	2	0.60	0.51	1	-0.20	4	0.22	0.41	0.12	+0.46	4	0.40	0.62	0.38	+0.29
**18**	24	8	0.88	0.79	0.85	-0.11	5	0.38	0.58	0.02	+0.35	2	0.42	0.52	0.67	+0.16	7	0.38	0.46	0.04	+0.14	4	0.42	0.54	0.06	+0.23
**19**	11	5	0.64	0.72	0.29	+0.05	4	0.45	0.71	0.33	+0.30	2	0.45	0.54	1	+0.00	2	0.10	0.19	1	-	4	0.27	0.54	0.013	+0.49
**20**	20	8	0.85	0.75	0.21	-0.14	4	0.35	0.67	0.01	+0.47	3	0.55	0.55	1	-0.06	4	0.25	0.36	0.04	+0.21	4	0.65	0.64	0.66	-0.02
**21**	16	3	0.81	0.69	0.20	-0.19	3	0.31	0.59	0.03	+0.45	2	0.63	0.51	0.61	-0.24	6	0.50	0.56	0.21	+0.04	3	0.38	0.50	0.02	+0.19
**22**	13	4	0.62	0.76	0.48	+0.13	3	0.27	0.58	0.04	+0.55	2	0.62	0.49	0.57	-0.26	4	0.42	0.44	1	-0.13	3	0.39	0.40	1	-0.13
**23**	17	5	0.77	0.72	0.80	-0.07	4	0.41	0.64	0.10	+0.30	2	0.69	0.50	0.16	-0.40	6	0.44	0.48	0.33	-0.01	4	0.31	0.52	0.08	+0.34
**24a**	11	3	0.36	0.65	0.08	+0.39	4	0.46	0.72	0.20	+0.34	3	0.36	0.50	0.60	+0.18	2	0.09	0.18	1	-	3	**0.18**	**0.65**	**0.003**	**+0.69**
**24b**	12	3	0.58	0.57	1	-0.03	4	0.33	0.63	0.03	+0.48	2	0.58	0.52	1	-0.13	4	0.33	0.50	0.56	+0.22	5	0.50	0.44	1	-0.16
**24c**	12	4	0.42	0.66	0.19	+0.33	3	**0.18**	**0.66**	**0.003**	+0.70	3	0.42	0.55	0.55	+0.26	4	0.42	0.42	0.39	+0.02	3	0.33	0.37	1	-0.11
**24d**	11	5	0.73	0.74	0.32	+0.02	3	0.46	0.57	0.07	+0.21	2	0.45	0.46	1	+0.00	4	**0.09**	**0.41**	**0.002**	+0.74	3	0.36	0.62	0.02	+0.36
**25**	13	5	0.52	0.59	0.48	+0.02	4	0.31	0.66	0.01	+0.52	2	0.42	0.60	0.59	+0.20	2	0.25	0.41	0.41	+0.28	4	0.46	0.58	0.64	+0.21
**26**	13	4	0.62	0.69	0.67	+0.11	3	0.08	0.29	0.04	+0.66	2	0.23	0.58	0.08	+0.56	2	0.08	0.15	1	-	2	0.54	0.55	1	-0.06
**27**	17	5	**0.94**	**0.77**	**0.006**	-0.23	2	0.31	0.47	0.27	+0.34	2	0.24	0.47	0.09	+0.46	4	**0.50**	**0.68**	**0.0003**	+0.24	2	0.13	0.18	1	-0.03
**28**	10	6	0.70	0.85	1	+0.13	4	0.30	0.73	0.02	+0.59	3	0.44	0.67	0.72	+0.30	4	0.33	0.56	0.14	+0.31	5	0.60	0.63	0.80	-0.06
**29**	24	4	0.35	0.44	0.27	+0.20	4	**0.29**	**0.77**	**<0.0001**	+0.62	2	0.67	0.50	0.11	-0.35	4	0.58	0.57	0.28	-0.07	2	0.50	0.48	1	-0.05
**30**	24	2	0.25	0.37	0.23	+0.26	3	**0.33**	**0.65**	**0.0007**	+0.46	2	0.33	0.45	0.34	+0.21	2	0.08	0.12	1	-0.02	2	0.33	0.34	1	+0.01
**31**	17	2	0.42	0.44	1	-0.03	3	0.41	0.52	0.05	+0.15	2	0.18	0.49	0.02	+0.62	3	0.19	0.24	1	-0.05	2	0.18	0.46	0.04	+0.57
**34**	26	3	0.27	0.32	0.10	+0.17	3	**0.31**	**0.53**	**0.006**	+0.40	2	0.15	0.18	1	-0.06	4	0.35	0.45	0.02	+0.18	3	0.04	0.15	0.02	+0.66
**35**	21	6	0.52	0.57	0.69	+0.04	3	**0.19**	**0.65**	**<0.0001**	+0.69	2	0.43	0.51	0.66	+0.16	4	0.33	0.34	1	-0.11	2	0.38	0.45	1	+0.09

N= number of specimens amplified, N_A= number of alleles, H_O, H_E=Observed and Expected heterozygosity, P=exact probability for expected Hardy Weinberg equilibrium conditions for each locus/population combination (Arlequin v2.1), M= monomorphic. F_{IS}= Weir & Cockerham (1984) (GENEPOP v3.4). **Values in bold** departures from HWE significant after Bonferroni correction (populations analysed at 9 loci k=9, p1=0.05/9, at 10 loci k=10, p1=0.05/10). See Table 2 for population details

(B) Summary of population microsatellite data per locus (LIST14-013, LIST14-021, LIST14-025, LIST14-037, LIST14-079).

	LIST14-013					LIST14-021					LIST14-025					LIST14-037					LIST14-079 ^				
**Pop**	**N_A**	**H_O**	**H_E**	**P**	**F_{IS}**	**N_A**	**H_O**	**H_E**	**P**	**F_{IS}**	**N_A**	**H_O**	**H_E**	**P**	**F_{IS}**	**N_A**	**H_O**	**H_E**	**P**	**F_{IS}**	**N_A**	**H_O**	**H_E**	**P**	**F_{IS}**
**1**	2	0.48	0.53	1.00	+0.06	2	0.15	0.35	0.03	+0.52	2	0.42	0.38	1	-0.11	2	0.48	0.50	1	-0.02	NA	-	-	-	-
**2**	2	0.65	0.54	0.36	-0.27	2	0.06	0.11	1	-	2	0.39	0.37	1	-0.21	2	0.44	0.40	0.53	-0.26	NA	-	-	-	-
**3**	2	0.38	0.36	1	-0.06	2	0.12	0.15	1	-0.04	2	0.19	0.24	0.37	+0.19	2	0.38	0.50	0.42	+0.23	NA	-	-	-	-
**4**	2	0.50	0.48	1	-0.05	3	0.10	0.37	0.06	+0.65	2	0.50	0.48	1	-0.05	2	0.70	0.56	0.52	-0.37	NA	-	-	-	-
**5**	2	0.54	0.53	0.74	-0.09	2	0.08	0.12	1	-0.03	3	0.56	0.51	0.88	-0.10	2	0.45	0.41	0.69	-0.11	NA	-	-	-	-
**6**	2	0.50	0.51	1	+0.02	3	0.13	0.17	1	-0.03	2	0.13	0.16	1	-0.05	3	0.38	0.52	0.33	+0.24	3	0.37	0.32	1	-0.18
**7**	2	0.67	0.51	0.55	-0.33	3	0.50	0.51	0.02	+0.02	2	0.17	0.24	1	-0.05	4	0.42	0.37	1	-0.12	2	0.17	0.24	1	-0.05
**8**	2	0.33	0.50	0.54	+0.24	1	M	M	M	M	3	0.27	0.41	0.45	+0.23	2	0.53	0.50	1	-0.17	NA	-	-	-	-
**9a**	2	0.71	0.55	0.27	-0.43	4	0.64	0.70	0.59	+0.07	3	0.43	0.36	1	-0.19	2	0.46	0.49	1	+0.07	NA	-	-	-	-
**9b**	2	0.36	0.52	0.32	+0.32	4	**0.21**	**0.77**	**0.0004**	+0.71	3	0.42	0.42	1	-0.17	2	0.69	0.51	0.28	-0.39	NA	-	-	-	-
**10**	2	0.23	0.48	0.16	+0.45	2	0.38	0.52	0.58	+0.19	2	0.23	0.21	1	-0.09	2	0.38	0.58	0.57	+0.25	NA	-	-	-	-
**13**	2	0.46	0.54	1	+0.11	1	M	M	M	M	3	0.27	0.56	0.02	+0.46	2	0.27	0.33	1	-0.11	NA	-	-	-	-
**16**	4	0.82	0.71	0.33	-0.15	3	0.55	0.56	1	+0.02	2	0.45	0.37	1	-0.25	3	0.45	0.65	0.75	+0.26	3	0.46	0.71	0.28	+0.29
**17**	3	0.50	0.55	1	+0.06	3	**0.20**	**0.55**	**0.003**	+0.60	2	0.40	0.44	1	+0.10	3	0.70	0.56	0.74	-0.26	3	0.5	0.68	0.08	+0.17
**18**	5	0.46	0.64	0.05	+0.25	3	0.58	0.63	0.41	+0.07	3	0.52	0.62	0.61	+0.11	3	0.67	0.55	0.20	-0.27	6	0.5	0.49	0.90	-0.09
**19**	3	0.46	0.60	0.28	+0.25	3	0.09	0.40	0.01	+0.77	3	0.55	0.45	1	-0.22	4	0.73	0.68	1	-0.08	3	0.64	0.48	0.64	-0.35
**20**	4	0.50	0.60	0.47	+0.18	3	0.40	0.38	1	-0.19	2	0.58	0.54	0.66	-0.15	4	0.67	0.57	0.7	-0.18	4	0.4	0.43	0.32	-0.03
**21**	4	0.69	0.56	0.16	-0.23	3	0.50	0.59	0.15	+0.15	3	0.75	0.59	0.20	-0.36	4	0.56	0.54	0.05	-0.07	5	0.75	0.61	0.72	-0.23
**22**	2	0.46	0.44	1	-0.04	2	0.08	0.15	-	-	2	0.69	0.47	0.21	-0.50	2	0.46	0.49	1	+0.07	3	0.33	0.37	1	-0.11
**23**	4	0.59	0.68	0.06	+0.07	3	0.47	0.53	0.36	+0.10	4	0.65	0.59	0.90	-0.09	4	0.53	0.57	0.60	+0.07	7	0.59	0.66	0.62	+0.11
**24a**	2	0.55	0.52	1	-0.05	3	0.36	0.56	0.25	+0.28	3	0.73	0.58	0.78	-0.26	3	0.64	0.57	1	-0.13	5	0.36	0.62	0.07	+0.37
**24b**	3	0.46	0.59	0.75	+0.16	3	0.17	0.51	0.01	+0.67	4	0.58	0.66	0.77	+0.12	3	0.58	0.56	1	-0.04	3	0.33	0.51	0.20	+0.35
**24c**	5	0.58	0.70	0.85	+0.09	3	0.75	0.56	0.41	-0.36	3	0.67	0.50	0.64	-0.39	2	0.50	0.55	1	+0.02	4	0.42	0.66	0.28	+0.30
**24d**	3	0.55	0.50	1	-0.26	3	0.73	0.54	0.35	-0.38	3	0.46	0.46	1	-0.16	3	0.89	0.58	0.08	-0.58	5	0.45	0.67	0.12	+0.27
**25**	3	0.54	0.54	1	+0.01	3	0.39	0.45	0.52	+0.02	4	0.75	0.58	0.88	-0.30	4	0.67	0.62	0.84	-0.14	4	0.69	0.71	0.66	-0.03
**26**	2	0.46	0.37	1	-0.26	3	0.69	0.66	0.89	-0.06	4	0.39	0.44	0.68	+0.14	2	0.38	0.39	1	-0.20	3	0.54	0.66	0.57	+0.19
**27**	2	**0.88**	**0.51**	**0.003**	-0.78	3	0.47	0.59	0.77	+0.12	3	0.47	0.52	1	+0.10	3	0.59	0.58	0.52	-0.01	4	0.35	0.36	1	-0.12
**28**	4	0.70	0.54	0.74	-0.33	2	0.60	0.56	1	-0.20	3	0.60	0.65	0.73	-0.07	2	0.60	0.57	1	-0.15	3	0.30	0.42	0.48	+0.29
**29**	3	0.44	0.52	0.78	+0.14	2	0.04	0.08	1	-	3	0.46	0.46	1	+0.01	4	0.48	0.48	1	-0.06	2	0.29	0.25	1	-0.15
**30**	2	0.52	0.46	0.66	-0.13	2	0.00	0.12	0.02	+1	3	0.50	0.51	0.15	+0.03	3	0.38	0.51	0.26	+0.22	1	M	M	M	M
**31**	2	0.29	0.34	0.54	+0.13	2	0.06	0.22	0.09	+0.65	2	0.06	0.12	1	-	2	0.53	0.40	0.28	-0.33	NA	-	-	-	-
**34**	2	0.13	0.23	0.21	+0.35	2	0.12	0.22	0.19	+0.35	4	0.08	0.15	0.05	+0.32	4	0.46	0.52	0.06	+0.12	NA	-	-	-	-
**35**	2	0.50	0.43	0.62	-0.17	4	0.24	0.26	0.35	+0.10	3	0.45	0.53	0.65	+0.08	2	0.43	0.35	0.53	-0.25	NA	-	-	-	-

N= number of specimens amplified, N_A= number of alleles, H_O, H_E=Observed and Expected heterozygosity, P=exact probability for expected Hardy Weinberg equilibrium conditions for each locus/population combination (Arlequin v2.1). F_{IS}= Weir & Cockerham (1984) (GENEPOP V3.4). **Values in bold** departures from HWE significant after Bonferroni correction, populations analysed (9 loci k=9, p1=0.05/9, at 10 loci k=10, p1=0.05/10). ^ LIST14-079 amplified in subset of populations. M= monomorphic, NA= not amplified. See Table 2 for population details
