# Supplementary material for: Molecular Genetics Reveal That Silvatic Rhodnius prolixus Do Colonise Rural Houses
Source: PLoS Negl Trop Dis. 2008 Apr 2;2(4):e210. doi: 10.1371/journal.pntd.0000210 (PMC2270345; doi:10.1371/journal.pntd.0000210)
Supplement: Table S3 — The pairwise comparison of 33 populations from six Venezuelan States at 9 microsatellite loci, FST values below diagonal (p-values above) (Arlequin v2.1). Values in bold significant after sequential Bonferroni correction k = 528, p1 = 0.05/528, p≤0.0001. See Table 2 for population details. (0.05 MB PDF) [file pntd.0000210.s003.pdf]

Table S3 The pairwise comparison of 33 populations from six Venezuelan States at 9 microsatellite loci, FST values below diagonal (p-values above) (Arlequin v3.1).

	Pop 1	Pop 2	Pop 3	Pop 4	Pop 5	Pop 9a	Pop 9b	Pop 10	Pop 13	Pop 35	Pop 8	Pop 6	Pop 7	Pop 31	Pop 29	Pop 30	Pop 33	Pop 16	Pop 17	Pop 18	Pop 19	Pop 20	Pop 21	Pop 23	Pop 22	Pop 24a	Pop 24b	Pop 24c	Pop 24d	Pop 26	Pop 25	Pop 27	Pop 28
**Pop 1**		0.02049	**0.00000**	0.02346	0.00020	0.00139	0.03069	**0.00000**	0.00505	0.02435	0.01643	**0.00000**	**0.00000**	**0.00000**	**0.00000**	**0.00000**	**0.00000**	0.09989	0.00564	**0.00000**	0.00455	**0.00000**	**0.00000**	**0.00000**	0.00158	0.10504	**0.00010**	0.05168	**0.00000**	**0.00000**	0.00129	**0.00010**	**0.00000**
**Pop 2**	0.0319		**0.00000**	0.00050	0.42461	**0.00000**	0.00020	**0.00000**	0.56925	0.08791	0.48946	**0.00000**	0.00277	**0.00000**	**0.00000**	**0.00000**	**0.00000**	0.00129	0.08267	**0.00000**	0.05306	0.00010	**0.00000**	**0.00000**	0.00109	0.00238	**0.00010**	0.02475	0.00040	**0.00000**	0.00881	**0.00000**	**0.00000**
**Pop 3**	0.1274	0.1997		0.02356	**0.00000**	**0.00000**	**0.00000**	**0.00000**	**0.00000**	**0.00010**	0.00010	**0.00000**	**0.00000**	**0.00000**	**0.00000**	**0.00000**	**0.00000**	0.00000	**0.00000**	**0.00000**	**0.00000**	**0.00000**	**0.00000**	0.00010	**0.00000**	0.00178	**0.00000**	**0.00000**	**0.00000**	**0.00000**	0.00030	**0.00000**	**0.00010**
**Pop 4**	0.0463	0.0853	0.0606		0.00149	0.00020	0.00168	**0.00000**	0.00050	0.03554	0.01525	**0.00000**	**0.00000**	0.00149	**0.00000**	**0.00000**	**0.00000**	0.00634	0.03069	0.00030	0.00366	0.00218	**0.00000**	0.00040	0.01040	0.41709	0.00089	0.00604	0.00069	**0.00000**	0.05772	0.00030	0.00713
**Pop 5**	0.0432	0.0016	0.1705	0.0710		**0.00000**	**0.00000**	**0.00000**	0.16028	0.00812	0.02921	**0.00000**	**0.00000**	**0.00000**	**0.00000**	**0.00000**	**0.00000**	0.00020	0.03277	**0.00000**	0.05118	0.00723	**0.00000**	0.00020	0.00267	0.01129	0.00119	0.00723	0.00050	**0.00000**	0.01307	**0.00000**	0.00030
**Pop 9a**	0.0633	0.1007	0.2617	0.1038	0.1222		0.99832	**0.00000**	**0.00000**	**0.00000**	**0.00000**	**0.00000**	**0.00000**	0.00010	**0.00000**	**0.00000**	**0.00000**	0.00317	0.00109	**0.00000**	0.00079	**0.00000**	**0.00000**	**0.00000**	**0.00010**	0.00297	**0.00000**	0.00584	**0.00000**	**0.00000**	**0.00000**	**0.00000**	**0.00000**
**Pop 9b**	0.0397	0.1001	0.2377	0.0962	0.1130	-0.0279		**0.00000**	**0.00000**	0.00099	0.00010	**0.00000**	0.00010	**0.00000**	**0.00000**	**0.00000**	**0.00000**	0.06960	0.00347	**0.00010**	0.00604	**0.00000**	**0.00000**	**0.00000**	**0.00000**	0.04495	**0.00000**	0.08781	0.00030	**0.00000**	**0.00000**	**0.00000**	0.00059
**Pop 10**	0.1665	0.1891	0.2921	0.2182	0.2011	0.2154	0.1977		**0.00000**	**0.00000**	**0.00000**	**0.00000**	**0.00000**	**0.00000**	**0.00000**	**0.00000**	**0.00000**	**0.00000**	**0.00000**	**0.00000**	**0.00000**	**0.00000**	**0.00000**	**0.00000**	**0.00000**	**0.00010**	**0.00000**	**0.00000**	**0.00000**	**0.00000**	**0.00000**	**0.00000**	**0.00000**
**Pop 13**	0.0611	-0.0024	0.2322	0.1397	0.0154	0.1328	0.1278	0.2406		0.12593	0.19345	**0.00000**	0.00426	**0.00000**	**0.00000**	**0.00000**	**0.00000**	0.00188	0.07861	**0.00010**	0.09306	0.00129	0.00030	0.00178	0.00168	0.00564	0.00475	0.05445	0.00040	**0.00000**	0.02515	**0.00000**	0.00050
**Pop 35**	0.0306	0.0202	0.1252	0.0444	0.0291	0.0937	0.0845	0.2286	0.0225		0.03604	**0.00000**	**0.00000**	**0.00000**	**0.00000**	**0.00000**	**0.00000**	0.00446	0.07583	0.00020	0.01554	0.00020	**0.00000**	0.00089	0.00020	0.00891	0.00099	0.17870	0.00099	0.00040+	0.07326	**0.00000**	0.00515
**Pop 8**	0.0436	0.0024	0.1635	0.0700	0.0298	0.1158	0.1149	0.1361	0.0217	0.0360		0.00020	0.00218	0.00436	**0.00000**	**0.00000**	**0.00000**	0.00129	0.21166	**0.00010**	0.04544	0.00040	**0.00000**	0.00010	0.05336	0.06029	0.00119	0.00842	0.01960	**0.00000**	0.07336	0.00010	0.00040
**Pop 6**	0.1207	0.0675	0.2993	0.1880	0.1205	0.1794	0.1789	0.1272	0.1148	0.1573	0.0719		0.00584	**0.00000**	**0.00000**	**0.00000**	**0.00000**	**0.00000**	**0.00000**	**0.00000**	**0.00010**	**0.00000**	**0.00000**	**0.00000**	**0.00000**	**0.00000**	**0.00000**	**0.00000**	**0.00000**	**0.00000**	**0.00000**	**0.00000**	**0.00000**
**Pop 7**	0.0988	0.0563	0.2624	0.1714	0.0830	0.1622	0.1458	0.1304	0.0715	0.1156	0.0820	0.0445		**0.00000**	**0.00000**	**0.00000**	**0.00000**	0.00129	0.01198	**0.00000**	0.00871	**0.00000**	**0.00000**	0.00010	0.00030	0.00574	0.00099	0.00149	0.00079	**0.00000**	0.00178	**0.00000**	**0.00000**
**Pop 31**	0.1411	0.1307	0.2277	0.0960	0.1563	0.1228	0.1455	0.1650	0.1940	0.1550	0.0713	0.1393	0.1927		**0.00000**	**0.00000**	**0.00000**	**0.00000**	0.00426	**0.00000**	**0.00010**	**0.00000**	**0.00000**	0.00010	0.00059	0.00218	**0.00000**	**0.00000**	0.00149	**0.00000**	**0.00000**	**0.00000**	**0.00000**
**Pop 29**	0.1381	0.0972	0.2569	0.1368	0.1022	0.1800	0.1822	0.2933	0.1114	0.1132	0.1356	0.1702	0.1104	0.2199		**0.00000**	**0.00000**	**0.00000**	0.00020	**0.00000**	**0.00010**	**0.00000**	**0.00000**	**0.00000**	**0.00000**	**0.00000**	**0.00000**	**0.00000**	**0.00000**	**0.00000**	**0.00000**	**0.00000**	**0.00000**
**Pop 30**	0.1587	0.1433	0.2900	0.2382	0.1098	0.2199	0.2054	0.3513	0.1050	0.1447	0.2046	0.2642	0.1559	0.3274	0.1472		**0.00000**	**0.00000**	**0.00000**	**0.00000**	**0.00010**	**0.00000**	**0.00000**	**0.00000**	**0.00000**	**0.00000**	**0.00010**	**0.00000**	**0.00000**	**0.00000**	**0.00000**	**0.00000**	**0.00000**
**Pop 33**	0.1958	0.1382	0.3953	0.3141	0.1440	0.2469	0.2432	0.4179	0.1435	0.1987	0.2087	0.2172	0.1732	0.3291	0.1872	0.1977		**0.00000**	**0.00000**	**0.00000**	0.00059	**0.00000**	**0.00000**	**0.00000**	**0.00000**	**0.00000**	**0.00000**	**0.00000**	**0.00000**	**0.00000**	**0.00000**	**0.00000**	**0.00000**
**Pop 16**	0.0274	0.0883	0.1604	0.0717	0.0897	0.0697	0.0415	0.2395	0.1103	0.0606	0.0975	0.1759	0.1227	0.1753	0.1319	0.1809	0.1819		0.07682	0.01307	0.10039	0.00089	0.00574	0.03703	0.00030	0.08969	0.00762	0.43164	0.00564	0.00020	0.03227	0.00614	0.01733
**Pop 17**	0.0604	0.0297	0.1728	0.0521	0.0350	0.0812	0.0777	0.1767	0.0377	0.0290	0.0210	0.0990	0.0597	0.0815	0.0940	0.1471	0.1712	0.0410		0.63429	0.73418	0.75596+	0.20434	0.45679	0.25601	0.31056	0.62578	0.85635	0.97515	0.00495	0.80279	0.01168	0.68746
**Pop 18**	0.0818	0.0814	0.1641	0.0843	0.0625	0.1074	0.0889	0.1792	0.0753	0.0617	0.0819	0.1565	0.0812	0.1546	0.1312	0.1180	0.1791	0.0419	-0.0038		0.23107	0.21008	0.24740	0.98584	0.00594	0.05168	0.74686	0.64954	0.19305	**0.00000**	0.21483	0.00376	0.62766
**Pop 19**	0.0614	0.0333	0.1947	0.0856	0.0280	0.0839	0.0755	0.2136	0.0335	0.0465	0.0463	0.1229	0.0614	0.1316	0.0818	0.0966	0.0916	0.0350	-0.0075	0.0124		0.79834	0.00881	0.33809	0.03950	0.13157	0.14088	0.69379	0.46401	0.00050	0.60578	0.00248	0.04792
**Pop 20**	0.0736	0.0569	0.1601	0.0556	0.0295	0.1086	0.0997	0.1946	0.0583	0.0556	0.0614	0.1466	0.0888	0.1292	0.0933	0.0983	0.1650	0.0596	-0.0096	0.0077	-0.0101		0.02208	0.50144	0.10256	0.09880	0.32611	0.26057	0.06049	**0.00000**	0.27908	0.00040	0.21869
**Pop 21**	0.1009	0.1104	0.2086	0.1174	0.0817	0.1448	0.1210	0.2384	0.0936	0.0797	0.1140	0.2010	0.1148	0.2101	0.1452	0.1254	0.2185	0.0607	0.0122	0.0066	0.0457	0.0247		0.31165	0.00129	0.00317	0.37868	0.13484	0.00376	0.00030	0.01069	0.00505	0.83982
**Pop 23**	0.0687	0.0785	0.1743	0.0803	0.0538	0.1013	0.0817	0.2186	0.0723	0.0562	0.0900	0.1671	0.0995	0.1683	0.1238	0.0953	0.1683	0.0310	0.0011	-0.0147	0.0072	-0.0010	0.0040		0.02000	0.05267	0.82061	0.89338	0.07286	**0.00000**	0.11890	0.01663	0.78705
**Pop 22**	0.0640	0.0573	0.1707	0.0546	0.0486	0.1083	0.1045	0.1402	0.0797	0.0769	0.0335	0.1046	0.0974	0.0739	0.1464	0.1510	0.2394	0.1006	0.0115	0.0418	0.0428	0.0166	0.0651	0.03208		0.15652	0.35046	0.03257	0.03356	**0.00000**	0.03366	0.00069	0.02455
**Pop 24a**	0.0273	0.0625	0.1079	0.0079	0.0461	0.0684	0.0512	0.1128	0.0828	0.0591	0.0426	0.1217	0.0786	0.0848	0.1144	0.1664	0.2413	0.0368	0.0149	0.0284	0.0332	0.0219	0.0582	0.0300	0.0204		0.19513	0.10880	0.06494	**0.00000**	0.42224	0.00822	0.06851
**Pop 24b**	0.0862	0.0849	0.1972	0.0961	0.0638	0.1214	0.1025	0.1741	0.0693	0.0808	0.0828	0.1363	0.0764	0.1564	0.1411	0.1024	0.2202	0.0685	-0.0035	-0.0057	0.0282	0.0066	0.0040	-0.0104	0.0066	0.0232		0.43520	0.21721	**0.00010**	0.10534	0.00584	0.70508
**Pop 24c**	0.0319	0.0384	0.1711	0.0694	0.0411	0.0509	0.0349	0.2040	0.0350	0.0157	0.0562	0.1320	0.0744	0.1440	0.1086	0.1019	0.1471	0.0024	-0.0160	-0.0033	-0.0055	0.0085	0.0149	-0.0137	0.0393	0.0304	0.0064		0.18612	0.00653	0.35670	0.01515	0.47728
**Pop 24d**	0.1046	0.0666	0.2072	0.0947	0.0738	0.1035	0.1033	0.1840	0.0739	0.0714	0.0454	0.1222	0.0757	0.0781	0.1344	0.1783	0.1932	0.0692	-0.0287	0.0119	0.0029	0.0224	0.0474	0.0211	0.0316	0.0352	0.0151	0.0151		**0.00000**	0.25760	0.00109	0.17691
**Pop 26**	0.1529	0.1676	0.1819	0.1752	0.1506	0.2043	0.1768	0.3335	0.1496	0.0854	0.1678	0.2984	0.1832	0.2727	0.2157	0.1929	0.2825	0.1042	0.0792	0.0663	0.0955	0.1095	0.0701	0.0858	0.1830	0.1321	0.1157	0.0607	0.0927		0.00050	**0.00000**	0.00059
**Pop 25**	0.0606	0.0447	0.1222	0.0360	0.0380	0.1062	0.0967	0.1869	0.0460	0.0249	0.0328	0.1242	0.0743	0.1090	0.0929	0.1501	0.1659	0.0457	-0.0128	0.0109	-0.0012	0.0069	0.0412	0.0175	0.0342	0.0068	0.0252	0.0066	0.0098	0.0816		0.00050	0.04158
**Pop 27**	0.0771	0.1150	0.2216	0.1261	0.1023	0.1436	0.1138	0.2240	0.1440	0.1054	0.1250	0.1726	0.1126	0.2084	0.1724	0.1893	0.2273	0.0581	0.0577	0.0456	0.0778	0.0752	0.0445	0.0345	0.0886	0.0647	0.0625	0.0452	0.0853	0.1316	0.0859		0.04802
**Pop 28**	0.0925	0.0974	0.2176	0.0824	0.0745	0.1112	0.0977	0.2444	0.1019	0.0677	0.0995	0.1934	0.1297	0.1693	0.1361	0.1472	0.2449	0.0577	-0.0066	-0.0032	0.0372	0.0105	-0.0137	-0.0105	0.0373	0.0390	-0.0063	0.0022	0.0167	0.0954	0.0368	0.0370	

Values in bold significant after sequential Bonferroni correction k=528, p1=0.05/528 p≤0.0001. See Table 2 for population details

Table S3 The standard deviation of P values (Arlequin v3.1).

	Pop 1	Pop 2	Pop 3	Pop 4	Pop 5	Pop 9a	Pop 9b	Pop 10	Pop 13	Pop 35	Pop 8	Pop 6	Pop 7	Pop 31	Pop 29	Pop 30	Pop 33	Pop 16	Pop 17	Pop 18	Pop 19	Pop 20	Pop 21	Pop 23	Pop 22	Pop 24a	Pop 24b	Pop 24c	Pop 24d	Pop 26	Pop 25	Pop 27	Pop 28
**Pop 1**		±0.0013	**±0.0000**	±0.0017	±0.0001	±0.0003	±0.0015	**±0.0000**	±0.0007	±0.0015	±0.0014	**±0.0000**	**±0.0000**	**±0.0000**	**±0.0000**	**±0.0000**	**±0.0000**	±0.0032	±0.0007	**±0.0000**	±0.0007	**±0.0000**	**±0.0000**	**±0.0000**	±0.0004	±0.0035	**±0.0001**	±0.0024	**±0.0000**	**±0.0000**	±0.0003	**±0.0001**	**±0.0000**
**Pop 2**			**±0.0000**	±0.0003	±0.0054	**±0.0000**	±0.0001	**±0.0000**	±0.0046	±0.0031	±0.0052	**±0.0000**	±0.0005	**±0.0000**	**±0.0000**	**±0.0000**	**±0.0000**	±0.0003	±0.0030	**±0.0000**	±0.0022	±0.0001	**±0.0000**	**±0.0000**	±0.0003	±0.0005	**±0.0001**	±0.0015	±0.0002	**±0.0000**	±0.0010	**±0.0000**	**±0.0000**
**Pop 3**				±0.0014	**±0.0000**	**±0.0000**	**±0.0000**	**±0.0000**	**±0.0000**	**±0.0001**	±0.0001	**±0.0000**	**±0.0000**	**±0.0000**	**±0.0000**	**±0.0000**	**±0.0000**	±0.0000	**±0.0000**	**±0.0000**	**±0.0000**	**±0.0000**	**±0.0000**	±0.0001	**±0.0000**	±0.0004	**±0.0000**	**±0.0000**	**±0.0000**	**±0.0000**	±0.0002	**±0.0000**	**±0.0001**
**Pop 4**					±0.0004	±0.0001	±0.0004	**±0.0000**	±0.0002	±0.0017	±0.0012	**±0.0000**	**±0.0000**	±0.0004	**±0.0000**	**±0.0000**	**±0.0000**	±0.0008	±0.0017	±0.0002	±0.0006	±0.0005	**±0.0000**	±0.0002	±0.0011	±0.0046	±0.0003	±0.0008	±0.0003	**±0.0000**	±0.0024	±0.0002	±0.0008
**Pop 5**						**±0.0000**	**±0.0000**	**±0.0000**	±0.0036	±0.0008	±0.0016	**±0.0000**	**±0.0000**	**±0.0000**	**±0.0000**	**±0.0000**	**±0.0000**	±0.0001	±0.0017	**±0.0000**	±0.0024	±0.0008	**±0.0000**	±0.0001	±0.0005	±0.0011	±0.0003	±0.0009	±0.0002	**±0.0000**	±0.0011	**±0.0000**	±0.0002
**Pop 9a**							±0.0004	**±0.0000**	**±0.0000**	**±0.0000**	**±0.0000**	**±0.0000**	**±0.0000**	±0.0001	**±0.0000**	**±0.0000**	**±0.0000**	±0.0006	±0.0003	**±0.0000**	±0.0003	**±0.0000**	**±0.0000**	**±0.0000**	**±0.0001**	±0.0005	**±0.0000**	±0.0007	**±0.0000**	**±0.0000**	**±0.0000**	**±0.0000**	**±0.0000**
**Pop 9b**								**±0.0000**	**±0.0000**	±0.0003	±0.0001	**±0.0000**	±0.0001	**±0.0000**	**±0.0000**	**±0.0000**	**±0.0000**	±0.0025	±0.0006	**±0.0001**	±0.0008	**±0.0000**	**±0.0000**	**±0.0000**	**±0.0000**	±0.0018	**±0.0000**	±0.0027	±0.0002	**±0.0000**	**±0.0000**	**±0.0000**	±0.0002
**Pop 10**									**±0.0000**	**±0.0000**	**±0.0000**	**±0.0000**	**±0.0000**	**±0.0000**	**±0.0000**	**±0.0000**	**±0.0000**	**±0.0000**	**±0.0000**	**±0.0000**	**±0.0000**	**±0.0000**	**±0.0000**	**±0.0000**	**±0.0000**	**±0.0001**	**±0.0000**	**±0.0000**	**±0.0000**	**±0.0000**	**±0.0000**	**±0.0000**	**±0.0000**
**Pop 13**										±0.0034	±0.0040	**±0.0000**	±0.0006	**±0.0000**	**±0.0000**	**±0.0001**	**±0.0000**	±0.0004	±0.0025	**±0.0001**	±0.0028	±0.0004	±0.0002	±0.0004	±0.0004	±0.0008	±0.0006	±0.0023	±0.0002	**±0.0000**	±0.0015	**±0.0000**	±0.0002
**Pop 35**											±0.0016	**±0.0000**	**±0.0000**	**±0.0000**	**±0.0000**	**±0.0000**	**±0.0000**	±0.0006	±0.0027	±0.0002	±0.0012	±0.0001	**±0.0000**	±0.0003	±0.0001	±0.0009	±0.0003	±0.0039	±0.0004	±0.0002	±0.0024	**±0.0000**	±0.0008
**Pop 8**												±0.0001	±0.0005	±0.0006	**±0.0000**	**±0.0000**	**±0.0000**	±0.0004	±0.0039	**±0.0001**	±0.0018	±0.0002	**±0.0000**	±0.0001	±0.0022	±0.0026	±0.0003	±0.0010	±0.0014	**±0.0000**	±0.0027	±0.0001	±0.0002
**Pop 6**													±0.0008	**±0.0000**	**±0.0000**	**±0.0000**	**±0.0000**	**±0.0000**	**±0.0000**	**±0.0000**	**±0.0001**	**±0.0000**	**±0.0000**	**±0.0000**	**±0.0000**	**±0.0000**	**±0.0000**	**±0.0000**	**±0.0000**	**±0.0000**	**±0.0000**	**±0.0000**	**±0.0000**
**Pop 7**														**±0.0000**	**±0.0000**	**±0.0000**	**±0.0000**	±0.0004	±0.0011	**±0.0000**	±0.0009	**±0.0000**	**±0.0000**	±0.0001	±0.0002	±0.0008	±0.0004	±0.0004	±0.0003	**±0.0000**	±0.0004	**±0.0000**	**±0.0000**
**Pop 31**															**±0.0000**	**±0.0000**	**±0.0000**	**±0.0000**	±0.0007	**±0.0000**	**±0.0001**	**±0.0000**	**±0.0000**	±0.0001	±0.0002	±0.0004	**±0.0000**	**±0.0000**	±0.0004	**±0.0000**	**±0.0000**	**±0.0000**	**±0.0000**
**Pop 29**																**±0.0000**	**±0.0000**	**±0.0000**	±0.0001	**±0.0000**	**±0.0001**	**±0.0000**	**±0.0000**	**±0.0000**	**±0.0000**	**±0.0000**	**±0.0000**	**±0.0000**	**±0.0000**	**±0.0000**	**±0.0000**	**±0.0000**	**±0.0000**
**Pop 30**																	**±0.0000**	**±0.0000**	**±0.0000**	**±0.0000**	**±0.0001**	**±0.0000**	**±0.0000**	**±0.0000**	**±0.0000**	**±0.0000**	**±0.0001**	**±0.0000**	**±0.0000**	**±0.0000**	**±0.0000**	**±0.0000**	**±0.0000**
**Pop 33**																		**±0.0000**	**±0.0000**	**±0.0000**	±0.0002	**±0.0000**	**±0.0000**	**±0.0000**	**±0.0000**	**±0.0000**	**±0.0000**	**±0.0000**	**±0.0000**	**±0.0000**	**±0.0000**	**±0.0000**	**±0.0000**
**Pop 16**																			±0.0028	±0.0013	±0.0030	±0.0003	±0.0008	±0.0017	±0.0002	±0.0030	±0.0009	±0.0052	±0.0007	±0.0001	±0.0017	±0.0008	±0.0012
**Pop 17**																				±0.0047	±0.0046	±0.0045	±0.0042	±0.0052	±0.0038	±0.0048	±0.0050	±0.0035	±0.0015	±0.0007	±0.0041	±0.0011	±0.0047
**Pop 18**																					±0.0041	±0.0037	±0.0038	±0.0011	±0.0007	±0.0023	±0.0045	±0.0046	±0.0039	**±0.0000**	±0.0042	±0.0006	±0.0055
**Pop 19**																						±0.0038	±0.0010	±0.0049	±0.0020	±0.0033	±0.0034	±0.0043	±0.0043	±0.0003	±0.0052	±0.0005	±0.0024
**Pop 20**																							±0.0014	±0.0042	±0.0032	±0.0029	±0.0041	±0.0041	±0.0021	**±0.0000**	±0.0044	±0.0002	±0.0043
**Pop 21**																								±0.0047	±0.0004	±0.0006	±0.0048	±0.0033	±0.0006	±0.0002	±0.0010	±0.0007	±0.0034
**Pop 23**																									±0.0015	±0.0021	±0.0044	±0.0032	±0.0026	**±0.0000**	±0.0032	±0.0012	±0.0036
**Pop 22**																										±0.0035	±0.0050	±0.0016	±0.0017	**±0.0000**	±0.0017	±0.0003	±0.0015
**Pop 24a**																											±0.0037	±0.0031	±0.0023	**±0.0000**	±0.0052	±0.0008	±0.0025
**Pop 24b**																												±0.0046	±0.0038	**±0.0001**	±0.0031	±0.0009	±0.0045
**Pop 24c**																													±0.0041	±0.0008	±0.0047	±0.0012	±0.0051
**Pop 24d**																														**±0.0000**	±0.0044	±0.0003	±0.0034
**Pop 26**																															±0.0002	**±0.0000**	±0.0002
**Pop 25**																																±0.0002	±0.0019
**Pop 27**																																	±0.0022
**Pop 28**																																	
