# Supplementary material for: Molecular Genetics Reveal That Silvatic Rhodnius prolixus Do Colonise Rural Houses
Source: PLoS Negl Trop Dis. 2008 Apr 2;2(4):e210. doi: 10.1371/journal.pntd.0000210 (PMC2270345; doi:10.1371/journal.pntd.0000210)
Supplement: Table S4 — The pairwise comparison of a subset of 20 populations at 10 microsatellite loci FST values below diagonal (p-values above) (Arlequin v2.1). Values in bold significant after sequential Bonferroni correction k = 190, p1 = 0.05/190, p≤0.0003. (0.04 MB PDF) [file pntd.0000210.s004.pdf]

Table S4 The pairwise comparison of a subset of 20 populations at 10 microsatellite loci FST values below diagonal (p-values above) (Arlequin v3.1).

	Pop 6	Pop 7	Pop 29	Pop 30	Pop 16	Pop 17	Pop 18	Pop 19	Pop 20	Pop 21	Pop 23	Pop 22	Pop 24a	Pop 24b	Pop 24c	Pop 24d	Pop 26	Pop 25	Pop 27	Pop 28
Pop 6		0.0065	**0.0000**	**0.0000**	**0.0000**	**0.0000**	**0.0000**	**0.0000**	**0.0000**	**0.0000**	**0.0000**	**0.0000**	**0.0000**	**0.0000**	**0.0000**	**0.0000**	**0.0000**	**0.0000**	**0.0000**	**0.0000**
Pop 7	0.0415		**0.0000**	**0.0000**	**0.0001**	**0.0003**	**0.0000**	**0.0001**	**0.0000**	**0.0000**	**0.0000**	**0.0000**	**0.0000**	**0.0002**	**0.0000**	**0.0000**	**0.0000**	**0.0000**	**0.0000**	**0.0000**
Pop 29	0.2357	0.2046		**0.0000**	**0.0000**	**0.0000**	**0.0000**	**0.0000**	**0.0000**	**0.0000**	**0.0000**	**0.0000**	**0.0000**	**0.0000**	**0.0000**	**0.0000**	**0.0000**	**0.0000**	**0.0000**	**0.0000**
Pop 30	0.3532	0.2962	0.1466		**0.0000**	**0.0000**	**0.0000**	**0.0000**	**0.0000**	**0.0000**	**0.0000**	**0.0000**	**0.0000**	**0.0000**	**0.0000**	**0.0000**	**0.0000**	**0.0000**	**0.0000**	**0.0000**
Pop 16	0.1873	0.1533	0.1365	0.2071		0.1048	0.0064	0.0987	**0.0003**	0.0061	0.0589	**0.0000**	0.1083	0.0104	0.5427	0.0070	**0.0000**	0.0094	0.0016	0.0110
Pop 17	0.1250	0.1032	0.0981	0.1713	0.0336		0.4004	0.7408	0.4637	0.2164	0.5846	0.1641	0.3740	0.6992	0.9282	0.8955	0.0011	0.3487	0.0054	0.4362
Pop 18	0.2093	0.1609	0.1241	0.1198	0.0470	0.0044		0.1959	0.2530	0.2100	0.8577	0.0065	0.0635	0.6867	0.3284	0.1857	**0.0000**	0.0117	0.0040	0.6615
Pop 19	0.1643	0.1266	0.0788	0.1110	0.0310	-0.0082	0.0126		0.7439	0.0114	0.3577	0.0340	0.1756	0.1869	0.7203	0.3955	**0.0000**	0.1231	0.0023	0.0400
Pop 20	0.2081	0.1751	0.0879	0.1007	0.0644	0.0010	0.0058	-0.0080		0.0144	0.2401	0.1360	0.1088	0.2831	0.0977	0.0529	**0.0000**	0.0077	**0.0002**	0.2701
Pop 21	0.2267	0.1655	0.1401	0.1376	0.0542	0.0098	0.0071	0.0398	0.0246		0.3757	0.0006	0.0059	0.4371	0.1367	0.0054	**0.0000**	0.0013	0.0025	0.7811
Pop 23	0.1861	0.1395	0.1231	0.1173	0.0259	-0.0026	-0.0077	0.0055	0.0062	0.0015		0.0132	0.0810	0.8524	0.9363	0.0671	**0.0000**	0.0235	0.0082	0.5543
Pop 22	0.1826	0.1929	0.1378	0.1535	0.1012	0.0183	0.0369	0.0391	0.0131	0.0604	0.0339		0.1904	0.3614	0.0163	0.0324	**0.0000**	0.0006	0.0006	0.0292
Pop 24a	0.1631	0.1399	0.1098	0.1761	0.0321	0.0117	0.0260	0.0262	0.0209	0.0498	0.0247	0.0181		0.2793	0.1447	0.0778	**0.0000**	0.1647	0.0077	0.0658
Pop 24b	0.1751	0.1377	0.1347	0.1158	0.0627	-0.0056	-0.0036	0.0224	0.0083	0.0016	-0.0111	0.0062	0.0179		0.4827	0.1904	**0.0000**	0.0137	0.0065	0.6113
Pop 24c	0.1496	0.1118	0.1122	0.1317	-0.0008	-0.0189	0.0064	-0.0060	0.0185	0.0140	-0.0147	0.0444	0.0262	0.0047		0.1323	0.0020	0.0835	0.0071	0.2181
Pop 24d	0.1799	0.1545	0.1336	0.1902	0.0644	-0.0184	0.0120	0.0055	0.0224	0.0410	0.0212	0.0320	0.0320	0.0171	0.0212		**0.0000**	0.0294	0.0007	0.2373
Pop 26	0.3114	0.2256	0.2223	0.2263	0.1040	0.0822	0.0795	0.1000	0.1206	0.0728	0.0845	0.1853	0.1262	0.1170	0.0669	0.0977		0.0007	**0.0000**	**0.0001**
Pop 25	0.1714	0.1399	0.1187	0.1927	0.0584	0.0075	0.0350	0.0219	0.0358	0.0506	0.0286	0.0599	0.0217	0.0425	0.0251	0.0300	0.0668		**0.0000**	0.0015
Pop 27	0.2348	0.2011	0.1626	0.1875	0.0665	0.0627	0.0422	0.0736	0.0693	0.0447	0.0394	0.0812	0.0615	0.0598	0.0523	0.0839	0.1431	0.1072		0.0449
Pop 28	0.2523	0.2125	0.1316	0.1523	0.0620	0.0061	-0.0040	0.0372	0.0082	-0.0104	-0.0012	0.0344	0.0377	-0.0010	0.0155	0.0134	0.1076	0.0601	0.0356	

Values in Bold significant after sequential Bonferroni correction k=190, p1=0.05/190 p≤0.0003

Table S4 The standard deviation of P values (Arlequin v3.1).

	Pop 6	Pop 7	Pop 29	Pop 30	Pop 16	Pop 17	Pop 18	Pop 19	Pop 20	Pop 21	Pop 23	Pop 22	Pop 24a	Pop 24b	Pop 24c	Pop 24d	Pop 26	Pop 25	Pop 27	Pop 28
Pop 6		±0.0008	**±0.0000**	**±0.0000**	**±0.0000**	**±0.0000**	**±0.0000**	**±0.0000**	**±0.0000**	**±0.0000**	**±0.0000**	**±0.0000**	**±0.0000**	**±0.0000**	**±0.0000**	**±0.0000**	**±0.0000**	**±0.0000**	**±0.0000**	**±0.0000**
Pop 7			**±0.0000**	**±0.0000**	**±0.0001**	**±0.0002**	**±0.0000**	**±0.0001**	**±0.0000**	**±0.0000**	**±0.0000**	**±0.0000**	**±0.0000**	**±0.0001**	**±0.0000**	**±0.0000**	**±0.0000**	**±0.0000**	**±0.0000**	**±0.0000**
Pop 29				**±0.0000**	**±0.0000**	**±0.0000**	**±0.0000**	**±0.0000**	**±0.0000**	**±0.0000**	**±0.0000**	**±0.0000**	**±0.0000**	**±0.0000**	**±0.0000**	**±0.0000**	**±0.0000**	**±0.0000**	**±0.0000**	**±0.0000**
Pop 30					**±0.0000**	**±0.0000**	**±0.0000**	**±0.0000**	**±0.0000**	**±0.0000**	**±0.0000**	**±0.0000**	**±0.0000**	**±0.0000**	**±0.0000**	**±0.0000**	**±0.0000**	**±0.0000**	**±0.0000**	**±0.0000**
Pop 16						±0.0030	±0.0009	±0.0030	**±0.0002**	±0.0007	±0.0024	**±0.0000**	±0.0026	±0.0010	±0.0053	±0.0009	**±0.0000**	±0.0010	±0.0004	±0.0011
Pop 17							±0.0046	±0.0042	±0.0048	±0.0034	±0.0045	±0.0035	±0.0052	±0.0044	±0.0026	±0.0032	±0.0003	±0.0043	±0.0007	±0.0052
Pop 18								±0.0040	±0.0041	±0.0040	±0.0036	±0.0007	±0.0020	±0.0047	±0.0048	±0.0044	**±0.0000**	±0.0012	±0.0007	±0.0047
Pop 19									±0.0046	±0.0010	±0.0048	±0.0016	±0.0041	±0.0042	±0.0043	±0.0047	**±0.0000**	±0.0028	±0.0005	±0.0021
Pop 20										±0.0010	±0.0040	±0.0038	±0.0029	±0.0043	±0.0028	±0.0023	**±0.0000**	±0.0008	**±0.0001**	±0.0046
Pop 21											±0.0051	±0.0002	±0.0007	±0.0052	±0.0031	±0.0008	**±0.0000**	±0.0004	±0.0005	±0.0044
Pop 23												±0.0012	±0.0025	±0.0035	±0.0025	±0.0027	**±0.0000**	±0.0014	±0.0010	±0.0048
Pop 22													±0.0034	±0.0046	±0.0013	±0.0019	**±0.0000**	±0.0002	±0.0002	±0.0018
Pop 24a														±0.0041	±0.0034	±0.0029	**±0.0000**	±0.0042	±0.0008	±0.0022
Pop 24b															±0.0048	±0.0038	**±0.0000**	±0.0011	±0.0009	±0.0048
Pop 24c																±0.0031	±0.0004	±0.0025	±0.0009	±0.0039
Pop 24d																	**±0.0000**	±0.0016	±0.0003	±0.0039
Pop 26																		±0.0003	**±0.0000**	**±0.0001**
Pop 25																			**±0.0000**	±0.0004
Pop 27																				±0.0022
Pop 28																				
